# Modeling of Mechanosensing Mechanisms Reveals Distinct Cell Migration Modes to Emerge From Combinations of Substrate Stiffness and Adhesion Receptor–Ligand Affinity

**DOI:** 10.3389/fbioe.2020.00459

**Published:** 2020-06-03

**Authors:** Diego A. Vargas, Inês G. Gonçalves, Tommy Heck, Bart Smeets, Laura Lafuente-Gracia, Herman Ramon, Hans Van Oosterwyck

**Affiliations:** ^1^Mechanical Engineering Department, MAtrix: Mechanobiology and Tissue Engineering, Biomechanics Division, KU Leuven, Leuven, Belgium; ^2^Mechanical Engineering Department, Multiscale in Mechanical and Biological Engineering, Aragon Institute of Engineering Research, University of Zaragoza, Zaragoza, Spain; ^3^Mechatronics Biostatistics and Sensors, Biosystems Department, Particulate Dynamics, KU Leuven, Leuven, Belgium; ^4^Prometheus: Division of Skeletal Tissue Engineering, KU Leuven, Leuven, Belgium

**Keywords:** cell migration, mesenchymal, mechanosensing, computational modeling, discrete element method, traction force

## Abstract

Mesenchymal cell migration is an integral process in development and healing. The process is regulated by both mechanical and biochemical properties. Mechanical properties of the environment are sensed through mechanosensing, which consists of molecular responses mediated by mechanical signals. We developed a computational model of a deformable 3D cell on a flat substrate using discrete element modeling. The cell is polarized in a single direction and thus moves along the long axis of the substrate. By modeling discrete focal adhesions and stress fibers, we implement two mechanosensing mechanisms: focal adhesion stabilization by force and stress fiber strengthening upon contraction stalling. Two substrate-associated properties, substrate (ligand) stiffness and adhesion receptor–ligand affinity (in the form of focal adhesion disassembly rate), were varied for different model setups in which the mechanosensing mechanisms are set as active or inactive. Cell displacement, focal adhesion number, and cellular traction were quantified and tracked in time. We found that varying substrate stiffness (a mechanical property) and adhesion receptor–ligand affinity (a biochemical property) simultaneously dictate the mode in which cells migrate; cells either move in a smooth manner reminiscent of keratocytes or in a cyclical manner reminiscent of epithelial cells. Mechanosensing mechanisms are responsible for the range of conditions in which a cell adopts a particular migration mode. Stress fiber strengthening, specifically, is responsible for cyclical migration due to build-up of enough force to elicit rupture of focal adhesions and retraction of the cellular rear. Together, both mechanisms explain bimodal dependence of cell migration on substrate stiffness observed in the literature.

## Introduction

Different cell types in the body have been found to migrate under different conditions and stimuli, usually confined to morphogenesis but also in healing and disease. There are multiple modes of cell migration, both individual and collective. One of the better understood is single-cell mesenchymal migration. This motility mode is characterized by a cyclic motion consisting of cell polarization, in which the cell forms a protrusive front, then forms new focal adhesions (FAs) to the substrate along the expanding front, and retracts its rear (Lauffenburger and Horwitz, 1996). Mesenchymal migration is mostly observed during developmental and wound-healing processes; however, it can be re-created *in vitro*. Cell types that display this migration type include fibroblasts, endothelial cells, and tumor cells (Kalluri and Weinberg, 2009), as well as keratocytes (Lee et al., 1993).

The protrusive front consists of an extension of the cell membrane through the polymerization of actin. FAs consist of multi-protein complexes that link the internal cell cytoskeleton with the ligands in the ECM outside of the cell; these complexes are responsive to physical forces. Finally, retraction occurs via contraction of internal actin stress fibers (SFs) via engaged myosin II molecular motors, pulling the rear forward when rear FAs disassemble (Hynes, 2009).

Cells are highly active systems that interact with their environment. Multiple studies have demonstrated that substrate stiffness affects the magnitude of tractions exerted on the substrate; in some cases, a stiffer substrate will result in higher tractions (Ghibaudo et al., 2008; Califano and Reinhart-King, 2010; Izquierdo-Álvarez et al., 2018), while in others the opposite is observed (Chan and Odde, 2008). This observation has been credited with resulting in cell migration speed also being affected by substrate stiffness (Pathak and Kumar, 2012). Some cells will migrate in the direction of a stiffer substrate in a process known as durotaxis (Lo et al., 2000).

The process by which a cell senses its mechanical environment is termed mechanosensing, a process increasingly studied experimentally (Friedrich et al., 2019; Mohammed et al., 2019). Mechanosensing enables a cell to respond to an external mechanical force (e.g., fluid shear forces) (Tzima et al., 2005; Tovar-Lopez et al., 2019) or to probe and interact with the mechanical properties (such as stiffness) of its environment (McCain et al., 2012; Polio et al., 2019). In the case of cell migration, mechanosensing plays a role when cells modulate the forces they generate based on substrate stiffness. The mechanisms responsible for this have been found to be molecular. First, an FA will be stabilized by force because talin, one of the component molecules in the complex that links the actin cytoskeleton to the transmembrane protein integrin and unfolds with force allowing the protein vinculin to bind and stabilize the complex (del Rio et al., 2009). With talin silenced, cells are not able to exert high tractions (Elosegui-Artola et al., 2016). Second, the actin cytoskeleton has been found to strengthen in its contractile force upon stalling (i.e., when the cell cannot further contract due to resistance from the ECM). Because deformation of a stiffer substrate requires more force, stalling and thus strengthening occur more often (Parameswaran et al., 2014; Müller and Pompe, 2016; Wolfenson et al., 2016).

Computational models have been used to study cell migration. They are a cheap alternative to experiments in which single parameters can be varied precisely. Models range from 1D models of molecular processes at the lamellipodium (Lp) (Dawes and Edelstein-Keshet, 2007) to 3D multiscale models that include intracellular cytoskeletal components or the ECM itself (Kim et al., 2018).

One of the most advanced models of mesenchymal migration (Kim et al., 2013) stands out in its consideration of cellular anatomy; it consists of a 3D cell with a deformable cortex, FA dynamics, lamellipodia protrusion, and cytoskeleton and nuclear remodeling, as well as actin contraction. Researchers varied ligand density and found that migration speed was maximal at a particular intermediate density.

Another relevant model, the clutch model, looks at mechanosensing at the adhesion level (Chan and Odde, 2008). By modeling a series of adhesions as clutches that connect a deformable ligand with a fiber pulled by molecular motors, this model simulated substrate deformation locally at an adhesion cluster and binding frequency as a function of individual adhesion binding dynamics and force sensitivity of adhesions (Bangasser et al., 2013). This model predicted an optimal (intermediate) value of substrate stiffness at which traction would be highest locally at the Lp. This work, however, could not conclude the effect of mechanosensing at the whole cell level and on migration.

We built upon these past models of cell migration and conceived one that looks at a whole cell while integrating its subcellular components responsible for mechanosensing. It is a computational model of a polarized deformable 3D cell based on mechanical principles, in which the cell is on a 2D substrate where ligand stiffness can be varied. We considered two mechanosensing mechanisms: FA maturation (*FA*_*mat*_) (i.e., stabilization of adhesion with force carried) and stress fiber strengthening (*SF*_*str*_) (i.e., increase in contraction force after stalling in fiber shortening). We varied two substrate-associated parameters, namely substrate stiffness and adhesion receptor–ligand affinity (in the form of FA disassembly rate). While the former presents a mechanical property, the latter can be influenced by chemical composition of the substrate surface, in particular, the type and number of adhesion ligand molecules present. Parameter variations were performed for four setups, defined by all possible combinations of the two considered mechanosensing mechanisms being ON or OFF, thus studying the effect of the mechanisms themselves and the interplay with substrate properties. The implementation of mechanosensing mechanisms separately and together reveal distinct sensitivities to parameters varied, which provides insights into cell migration itself.

To our knowledge, it is the first model of cell migration to include these mechanosensing mechanisms. Although Kim et al. modeled discrete FAs and SFs (Kim et al., 2013), there was no *FA*_*mat*_, but rather a weakening of adhesions with force (in the form of Bell's model [Bell, 1978]). Similarly, the Kim et al. model is detailed in its implementation of actin and myosin dynamics leading to SF stalling (i.e., a target speed rather than force was implemented, to mimic myosin walking), but there was no strengthening of SFs. Additional implementation of a rupture force for FAs as in the clutch model revealed that retraction of the rear in mesenchymal migration can occur via collective failure of multiple FAs, reminiscent of the load-and-fail cycles achieved in the clutch model for single adhesion clusters (Chan and Odde, 2008). This process could account for cyclic retraction of the rear, thus defining the migration mode adopted by a cell and its speed in many conditions. We also found that the implemented mechanosensing mechanisms accounted for bimodal dependence of cellular tractions on substrate stiffness. Finally, the use of both mechanisms brings simulated cellular tractions closer to physiological values.

## Methods

The system modeled consisted of a 3D cell placed on a rigid substrate plane. The discrete element method (DEM) was used to represent both cell and substrate via lattice-free nodes, connected to each other in such a way that each body (i.e., cell and substrate plane) consisted of a mesh of triangular elements where the nodes are the vertices of the triangles. The triangles were used to calculate mechanical interactions (contact forces) between bodies (in this case between cell and substrate), as presented in previous work studying cell spreading (Odenthal et al., 2013) and cell–cell separation (Smeets et al., 2019). New elements to study cell migration are highlighted in the Methods section, as well as components considered crucial to mechanosensing and migration; otherwise, a summary is provided and detailed information presented in the Supplementary Material. Model parameters are listed along with their values and source of estimation in Table 1.

**Table 1:** Parameters for model implementation.

**Symbol**	**Parameter**	**Value**	**Units**	**Source**
***R*_*c*_**	cell radius	8	μm	–
$L_{fib,min}^{0}$	minimum initial fiber length	8	μm	–
***k*_*cortex*_**	stiffness cell cortex	2.9 × 10^−4^	N/m	Pontes et al., 2017
****Λ**^*d*^**	cortex damping	5 × 10^−1^	N·s/m	trial runs
***k*_*gen*, **Δ***i*_**	actin generation rate	1.18 × 10^11^	1/m^2^/s	Delorme et al., 2007; Fischer et al., 2009
***k*_*gen*_**	actin degradation rate	0.016	1/s	Delorme et al., 2007; Fischer et al., 2009
***D*_*actin*_**	actin diffusion constant in cortex	8 × 10^−14^	1/s	Fischer et al., 2009
***k*_*FA*_**	stiffness focal adhesion (FA)	1 × 10^3^	N/m	Bangasser et al., 2013
***k*_*ECM*_**	stiffness substrate ligand	range(0.001,0.5)	N/m	Bangasser et al., 2013
***L*_*FA*, 0_**	resting length FA	0.1	nm	trial runs
***L*_*KCM*, 0_**	resting length ligand	1.4	nm	trial runs
***r*_*on, FA*_**	binding rate FA	5 × 10^−3^	1/s	trial runs
$r_{on,FA}^{0}$	disassembly rate FA at zero force	range(5 × 10^−4^,5 × 10^−2^)	1/s	Webb et al., 2004; Berginski et al., 2011
****ζ**_*FA*_**	mechanosensing parameter FA	1.21	1/nN	trial runs
****λ**_*ref*_**	refractory period	30	min	trial runs
***k*_*prot*_**	proportionality protrusion	6 × 10^−10^	m^2^	trial runs
***F*_*am*_**	reference actomyosin force	1.0	nN	Dembo and Wang, 1999; Moore et al., 2010
***F*_*rup*_**	FA rupture force	7.7	nN	Balaban et al., 2001
***L*_*thr*_**	stress fiber shortening threshold	7.5 × 10^−5^	μm	trial runs
**μ**	Slope of force drop with SF shortening	5.0 × 10^6^	μm	trial runs
****Δ***L*_50_**	Δ*L*_*fib*_ leading to 50% force drop	3.5 × 10^−6^	μm	Sato et al., 2005
***n*_*thr*_**	FA rupture threshold	15	−	trial runs

**Figure 1:**
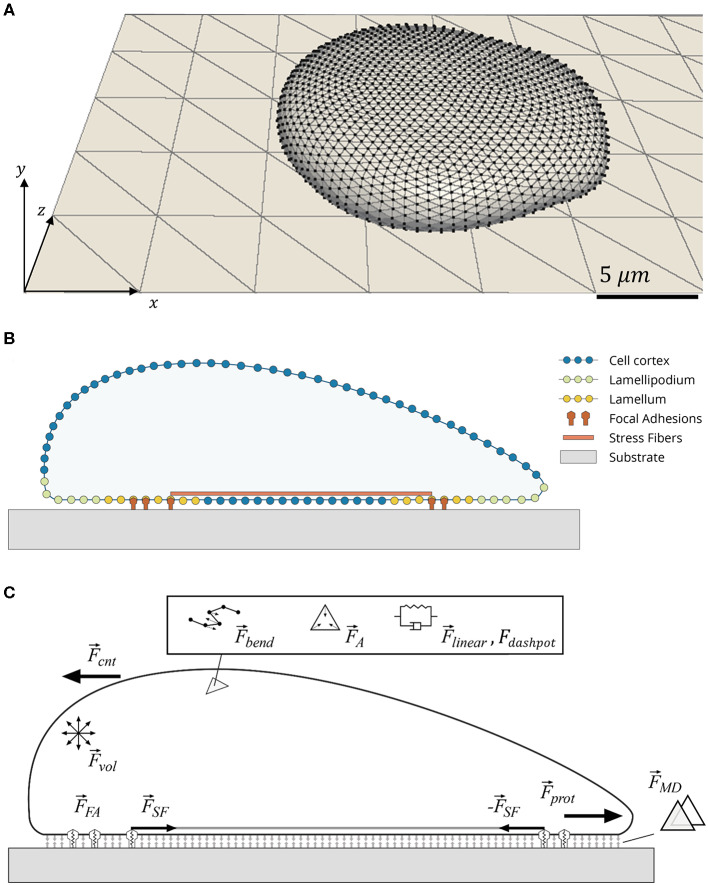
Schematic of cell model. **(A)** Tessellation of cell surface and substrate plane using triangles. Cell is a 3D object bound to a 2D substrate plane. **(B)** Cross-section of cell showing distinct cellular parts used in cell migration. **(C)** Forces involved in evolution of the mechanical system. The following relevant forces are indicated: cortical elastic spring (${\vec{F}}_{linear}$), cortical dissipation (${\vec{F}}_{dashpot}$), cortical bending (${\vec{F}}_{bend}$), local triangle and global cell area conservation (${\vec{F}}_{A}$), cell volume conservation (${\vec{F}}_{vol}$), membrane contact (${\vec{F}}_{MD}$), focal adhesion (${\vec{F}}_{FA}$), protrusion lamellipodium force (${\vec{F}}_{prot}$), internal counter force (${\vec{F}}_{cnt}$), and stress fiber (${\vec{F}}_{SF}$).

The cell is allowed to attach and spread on the substrate plane in an initialization phase, before FAs are allowed to form, mechanosensing mechanisms are activated, and migration occurs. This initialization phase is not considered in the analysis and was identical for all simulations ran. Once the system was initialized (a graphical representation of the system, cell and substrate plane, is presented in Figure 1A), specific parameters defining the different study conditions are changed, migration mechanisms activated, and evolution of the system recorded. For this reason, the data here presented corresponds to cells that always have a cell–ECM interface and are migrating from the start. The cell was programmed to be polarized and tracked over a simulated period of 24 h. Displacement of the cell body, traction on the substrate, and adhesion of the cell were tracked over time. A flowchart showing the main commands and their order of execution in the simulation loop is presented in Figure S6.

### Deformable Cell Model

The cell with radius *R*_*c*_ consisted of a triangular mesh representing the actin cortex; the cell is modeled as an empty deformable sphere with nodes being able to move in 3D (i.e., along *x, y, z* axes). The connections between nodes were viscoelastic Kelvin-Voigt elements (i.e., an elastic spring and viscous damper in parallel): The linear force arising from deformation of the line elements is denominated ${\overset{⇀}{F}}_{linear}$, and its magnitude is calculated according to the following equation:

(1) $$\begin{array}{r} {|\vec{F}}_{linear}|=\;k_{cortex}\left(d_{ij}-d_{ij}^{*}\right) \end{array}$$

where *d*_*ij*_ and $d_{ij}^{*}$ are the actual distance and equilibrium distance between nodes *i* and *j* (vertices), and *k*_*cortex*_ is the spring constant of the cellular cortex. Meanwhile damping by the dashpot element is described by ${\overset{⇀}{F}}_{dashpot}$, and its magnitude is calculated according to the following equation:

(2) $$\begin{array}{r} |{\vec{F}}_{dashpot}|=-\Lambda^{d}{\vec{n}}_{ij}\cdot {\vec{v}}_{ij} \end{array}$$

where Λ^*d*^ is the damping constant and ${\vec{n}}_{ij}\cdot {\vec{v}}_{ij}$ the projection of the velocity along the connecting axis between nodes *i* and *j*.

Additional forces defining geometry include local triangle and global cell area conservation (${\overset{⇀}{F}}_{A}$), a cell volume conservation (${\overset{⇀}{F}}_{vol}$), and a resistance to bending based on the angle between the two planes defined by neighboring triangles (${\overset{⇀}{F}}_{bend}$). These four forces ensure the cell maintains its relative rounded shape, its size does not vary wildly, and it does not extend infinitely while its front protrudes. A mathematical description of these forces is in the Supplementary Material (section Cortex Elasticity).

A general note for parameter selection is that, with this being a multiscale model (i.e., with cellular and subcellular scales), it is difficult to set every parameter equal to an experimental measurement: Many subcellular elements (e.g., organelles) were not included, and a series of simplifications needed to be made to model an active cytoskeleton. For parameters determining cortex mechanical properties, values were chosen based on a previous DEM model by the authors of a single cell spreading on a surface (Odenthal et al., 2013). That study analyzed spreading of a red blood cell over a shorter time scale. For this reason, we had to change certain parameters to include the additional subcellular elements, hence the statement “trial runs” for multiple parameters in Table 1.

### Substrate Plane

The substrate was modeled as a rigid plane, subdivided into right isosceles triangles with area of 2.102 μm^2^. Triangles on the plane were used to quantify traction by summing force exerted by the cell on each triangle with a defined area; this way, traction maps were generated (details in section Output metric calculation). Stiffness perceived by the cell arose from springs used to represent ligand molecules with a specific stiffness (*k*_*ECM*_) as part of FAs and discussed in section Adhesion.

### Cell Anatomy

Distinct functional cellular regions were defined using the triangles constituting the cellular cortex, specifically a lamellipodium (Lp), a front lamellum (Lm), and a rear Lm. The Lp is the outer most area in the leading front of the cell, where a protrusion force can be exerted outwards at the cellular front. Right behind where the Lp ends, there is a front Lm. At the rear another Lm can be defined. These lamella are where adhesions bound by SFs can mature into FAs (Delorme et al., 2007). A representation of the Lp and lamella of the simulated cell can be found in Figure S2. Beyond these two areas, the rest of the cell-substrate interface is a part of the cell body in which no protrusion or formation of FAs could occur.

To demarcate Lp and Lm, the approximate distance from the edge of the cell was determined at every time step. This was done by simulating the diffusion of globular actin at each triangle *i* (*G*_Δ*i*_) (units of molecules/μm^2^) and using thresholds in this concentration to determine where Lp and Lm begin and end. As the cell perimeter is the source of actin retrograde flow (Gardel et al., 2008), triangles *i* in the cell periphery acted as a source (generation rate *k*_*gen*, Δ*i*_ [1/μm^2^/s]), while all other triangles acted as a sink (degradation rate *k*_deg_ [1/s]). This effectively creates a gradient of actin concentration that is highest at the cell periphery and decays along the bottom (i.e., cell–substrate interface) and top (i.e., opposite) surfaces of the cell. The evolution in concentration of actin at each triangle is given according to:

(3) $$\begin{array}{r} \frac{\delta {\left[G\right]}_{\Delta i}}{\delta t}=k_{gen,\Delta i}-k_{deg}{\left[G\right]}_{\Delta i}+D_{actin}\nabla^{2}{\left[G\right]}_{\Delta i} \end{array}$$

where *D*_*actin*_ represents the diffusion constant of globular actin through the cell cortex. Thus, the third term in the right-hand side of the equation corresponds to diffusion across the cell surface (i.e., across the sides of the triangular element *i* in the 2D mesh) according to Fick's second law. For more information on how evolution of Lp and lamella were implemented, see the Supplementary Material.

### Migration Cycle

The cell was polarized in a single direction (x-axis in Figure 1A). Polarization was implemented in two ways: first, applying the protrusive force (${\vec{F}}_{prot}$) at the cell front; and second, creating an imbalance in disassembly probabilities in the front and rear of the cell. For an explanation of how the front and rear were defined, see (Figure S1).

### Protrusion

${\vec{F}}_{prot}$ was applied to in individual triangles in the Lp (located in the leading front of the cell), with direction and magnitude determined by [*G*]_Δ*i*_. The force acting on triangle *i* is represented by ${\vec{F}}_{prot,\Delta i}$:

(4) $$\begin{array}{r} {\vec{F}}_{prot,\Delta i}=\frac{k_{prot}{\left[G\right]}_{\Delta i}}{\left|{\nabla \left[G\right]}_{\Delta i}\right|}{\nabla \left[G\right]}_{\Delta i} \end{array}$$

where ∇[*G*]_Δ*i*_ is the gradient in concentration across triangles and *k*_*prot*_ a proportionality constant.

Biologically, protrusion force extends the membrane and is significantly lower than the force exerted by SFs responsible for retraction. In simulations, the average $\left|{\vec{F}}_{prot}\right|$ value per triangle was approximately 0.12 nN. Indeed, this value neared experimentally observed maxima for a comparable area (0.15 nN) (Gardel et al., 2008) and is one order of magnitude lower than that exerted at a FA by a SF.

Protrusion should not solely lead to migration, because internal forces in the cell should be balanced. For this reason, a force counter to protrusion was set up to act on the cell body. It acts on all cell nodes. Equation 5 describes the counter force (${\vec{F}}_{cnt}$) acting on each node *j*:

(5) $$\begin{array}{r} {\vec{F}}_{cnt,j}=\frac{-1}{n_{node}}\sum_{i=1}^{n_{\Delta ,Lp}}{\vec{F}}_{prot,\Delta i} \end{array}$$

where *n*_Δ, *Lp*_ is the number of triangles belonging to the Lp, and *n*_*node*_ the number of all nodes constituting the cell cortex.

Because the Lp fans out the leading front (Figure S1), the average value of ${\vec{F}}_{prot}$ is a vector close to the positive *x* direction (i.e., long axis of the substrate plane in the direction of motion). Thus, ${\vec{F}}_{cnt,j}$ points close to the negative *x* direction. As a result, the top of the cell (i.e., nodes not in cell–substrate interface) get pulled back. This gives the cell its characteristic shape with a thin front and thick rear observed in Figure 1.

### Adhesion

Two types of interactions between cells and substrate were modeled: transient and multi-protein complexes. The former represents transient binding of integrin molecules on the cell surface with ligands on the substrate. The latter represents FAs.

Transient adhesion between cell and substrate triangles that are in contact was modeled according to Maugis-Dugdale theory (Maugis, 1992), which accounts for adhesive contact mechanics. The local contact force (${\vec{F}}_{MD}$) between two contacting triangles was computed from the corresponding contact between two adhesive elastic spheres fitted to the two triangles. To achieve this, the local curvature of each of the triangles in the surface was calculated based on node coordinates, and a unique sphere is fit to each triangle (with the radius of the sphere corresponding to the flat substrate being infinite). An in-depth description of implementation can be found in the Supplementary Material (section Contact Mechanics).

FAs were modeled as discrete elements at the cell nodes, implemented as a system of two springs in series (Schwarz et al., 2006): A stiff spring represents the FA (*k*_*FA*_), while a softer spring represents the underlying ligand molecule (*k*_*ECM*_). The force carried by the two-spring system and thus the FA *i* (${\vec{F}}_{FA,i}$) is described by the following equation:

(6) $$\begin{array}{l} {\vec{F}}_{FA,i}=\{\begin{array}{ll} {\left(\frac{1}{k_{FA}}+\frac{1}{k_{ECM}}\right)}^{-1}\left(\left(L_{FA,0}+L_{ECM,0}\right)-L\right){\vec{n}}_{ji} & iff\;\left(\left(L_{FA,0}+L_{ECM,0}\right)-L\right)<0\\ 0\;{\vec{n}}_{ji} & iff\;\left(\left(L_{FA,0}+L_{ECM,0}\right)-L\right)\geq 0 \end{array} \end{array}$$

where *L*_*FA*, 0_ and *L*_*ECM*, 0_ are the equilibrium lengths of the FA and ECM ligand fiber, and *L* is the length at each corresponding time step. ${\vec{n}}_{ji}$ is the unit vector in the axis that runs from point *j* in the substrate (not necessarily a node) to cell node *i*. As *k*_*FA*_ ≫ *k*_*ECM*_, the FA force response is dictated by *k*_*ECM*_. This assumes that each FA only senses the substrate with which it is directly in contact. In this way different stiffness conditions can be modeled despite using a rigid plane as the substrate.

Any node in the Lm could form a FA with any point on the substrate as long as it was within a (vertical) distance of 0.075 μm. Formation of a FA was a stochastic process described by a binding rate (*r*_*on, FA*_). Similarly, a rate dictated how often FAs disassembled (*r*_*off, FA*_), thus describing adhesion receptor–ligand affinity. Because FAs were mechanosensitive, *FA*_*mat*_ was modeled by making *r*_*off, FA*_ dependent on the magnitude of the force carried by the FA. Based on modeling of catch bonds (Bangasser et al., 2013), Equation 7 describes the relation between disassembly rate and force carried by the FAs:

(7) $$\begin{array}{r} r_{off,FA,i}=\;\beta \;r_{off,FA}^{0}\;e^{-\zeta_{FA}|{\vec{F}}_{FA,i}\;|} \end{array}$$

**Figure 2:**
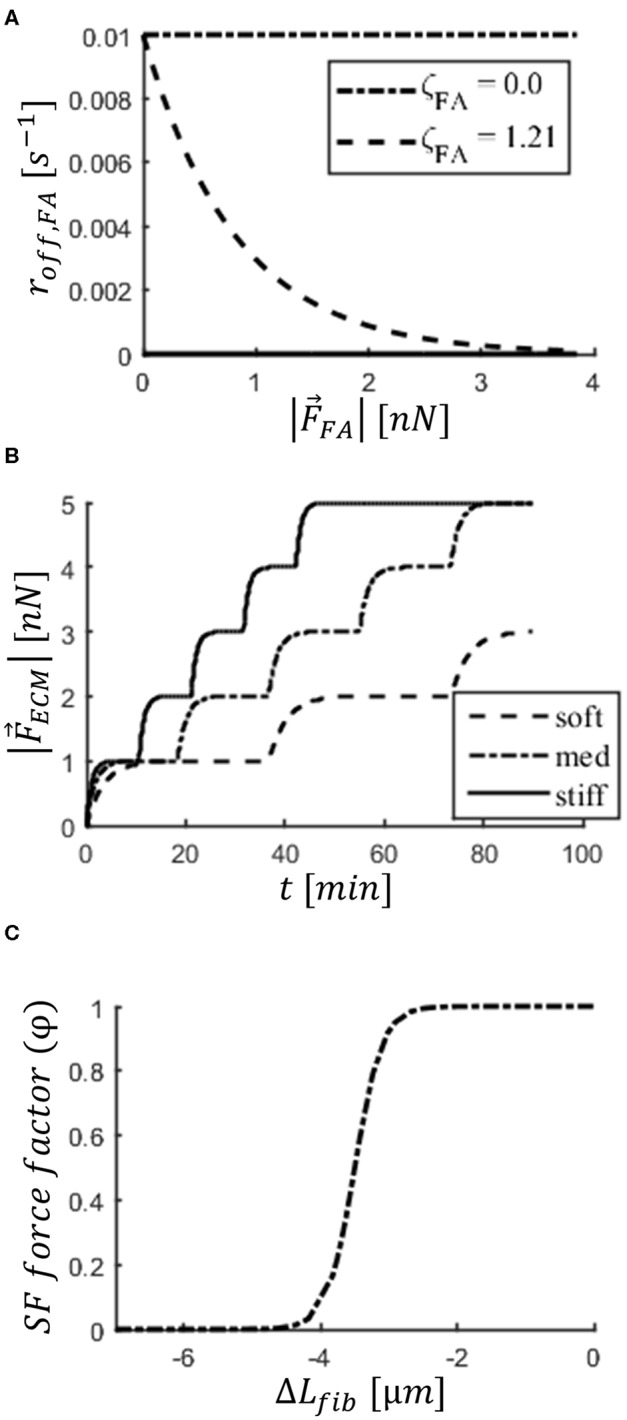
Implementation of mechanosensing mechanisms in the cell. **(A)** Focal adhesion (FA) disassembly rate (*r*_*off, FA*_) decreases with force carried by FA (*|*${\vec{F}}_{FA}|$). Two values of parameter (ζ_*FA*_) are displayed to demonstrate effect of focal adhesion maturation (*FA*_*mat*_). **(B)** Step increase in force carried by ECM (*|*${\vec{F}}_{ECM}|$) bound to a FA and applied by a stress fiber (SF). Steps correspond to stalling in SF contraction. The stiffer the substrate, the faster a SF strengthens, taking more steps during its lifetime. Graphs shown are a result of a simulation of an isolated two-spring system representing the FA and ligand molecule (ideal case). Substrate stiffness values shown: soft (1.2 × 10^−2^ N/m), med (2.4 × 10^−2^ N/m), stiff (4.16 × 10^−2^ N/m). **(C)** Dependence of factor φ on change in length of SFs (Δ*L*_*fib*_). The factor is multiplied by SF force (${\vec{F}}_{SF}$), implementing a weakening with strain (i.e., SF shortening).

$r_{off,FA}^{0}$ is the rate of disassembly when a FA carries no force, and ζ_*FA*_ indicates the degree of mechanosensing. β was used to vary the rate based on whether the FA is in the front (β = 1) or the rear (β = 2) of the cell to implement cell polarization; this factor makes sure FAs in the rear of the cell are more unstable than those in the front (Kim et al., 2013; Zhang et al., 2014). Figure 2A shows the dependence of $r_{off,FA}^{0}$ on $\left|{\vec{F}}_{FA}\right|$. For each $r_{off,FA}^{0}$, an expected FA lifetime (${\langle \lambda \rangle }_{FA}^{0}$) can be calculated according to:

(8) $$\begin{array}{r} {\langle \lambda \rangle }_{FA}^{0}=\;\frac{-1}{\ln\left(1-r_{off,FA}^{0}\right)} \end{array}$$

Equation 8 is derived from the assumption of FA dissociation as a Poisson process, with probability of dissociation being the cumulative distribution function. An explanation of this relation between disassembly rate and expected lifetime is available in the Supplementary Material (subsection Disassembly Rate and Expected Lifetime).

Additionally, if a FA was outside of the Lm whether in the Lp or cell body (e.g., due to displacement of the cell nodes with force or change in shape of the Lm) the force dependent rate of disassembly (*r*_*off, FA*_) was increased by a factor of 10 to ensure it is short-lived.

Finally, FAs could also rupture due to excessive force ($|{\vec{F}}_{FA}|>F_{rup}$). When a FA ruptured, the node where the FA was located underwent a refractory period (λ_*ref*_). During this period no new FA could form. It represented the time it takes for protein components of a FA lost in rupture to be recruited such that a new FA can form and bind the cytoskeleton (Fuhrmann and Engler, 2015; Winklbauer, 2015). For the remainder of this work, the term disassembly will be used exclusively to refer to stochastic removal of FAs, and the term rupture will be used exclusively to refer to removal of FAs due to excessive force acting on the adhesion.

### SF Formation, Contraction, and Strengthening

SFs were simulated as a pair of equal and opposite forces attracting a pair of cell nodes. The forces represented tension carried by SFs arising from the action of myosin II motors sliding along antiparallel actin bundles. A SF would form, connecting two FAs at random, if the potential fiber met two conditions:

1) It connected FAs that were not already bound to a SF.
2) It had a minimum initial length ($L_{fib,min}^{0}$).

Condition 2 is based on observations in the literature that fibers will only form along the long axis (but not the short axis) of a rectangular underlying pattern (McCain et al., 2012; Polio et al., 2019). The initial SF length ($L_{fib}^{0}$) is determined by the random pair of FAs that are connected. There is a minimum initial SF length ($L_{fib,min}^{0}$), but fibers can initially be longer and are allowed to shorten below this value.

Once formed, a SF remained bound to the cortex (i.e., cell nodes) and exerts force as long as there was at least at one of the nodes a FA adhering the node to the substrate; as soon as both FAs disassembled or ruptured, the SF was deleted (along with the corresponding force). A visualization of the location of FAs within the Lm and SFs can be found in the (Figure S2).

The second mechanosensing mechanism considered was *SF*_*str*_: a stepwise strengthening of SFs upon stalling of underlying substrate deformation. This mechanism is based on experimental and theoretical findings (Parameswaran et al., 2014; Wolfenson et al., 2016). The interval between maturation steps depended on substrate stiffness, with strengthening occurring more often on stiffer substrates, explaining how cells exert higher tractions on stiffer substrates. This behavior arises from the combination of the stiffness of an elastic spring representing the ligand molecule being stretched and the damping due to friction during motion, effectively behaving as a Kelvin-Voigt model (Schwarz et al., 2006).

Implementation of *SF*_*str*_ consisted in incrementing the magnitude of the force exerted by a SF on node *i* (${\overset{⇀}{F}}_{SF,i}$) by a factor (*n*_*str, f*_) every time the SF stalled. An average SF length (〈*L*_*fib*_〉) was calculated at every time step using a moving average filter with a time window of 10 s; the resulting average length is stored and compared every 10 s. Filtering ensures that the values compared are not affected by fluctuations in length due to the different forces acting on each node. A SF is said to stall the moment when the difference between subsequent length measurement (i.e., 〈*L*_*fib*_〉 measurements 10 s apart) fell below a threshold value (*L*_*thr*_).

The force exerted by SF *f* on node *i*, when connecting nodes *i* and *j*, is described by:

(9) $$\begin{array}{r} {\vec{F}}_{SF,i}=\;n_{str,f}\;F_{am}\;\varphi \left(\Delta L_{fib}\right)\;{\vec{n}}_{ij} \end{array}$$

For the factor *n*_*str, f*_, *f* is an index to refer to a particular SF. ${\vec{n}}_{ij}$ is the unit vector in the axis that runs from node *i* to node *j*. The factor φ is a function of Δ*L*_*fib*_ describing weakening of SFs with shortening and explained in the next subsection. Upon formation (φ = 1), SFs have a value of *n*_*str, f*_ = 1 and thus a force magnitude of *F*_*am*_. With each stalling event, the factor *n*_*str, f*_ was increased by the value of 1, up to 5 (a limit of 5 is set to avoid unlimited force exertion). An example of SF strengthening on substrates of three different stiffness values is shown in Figure 2B; this is an idealized case, presented for clarity, in which a node bound to the substrate plane via a two-spring system (representative of ligand molecule and FA), but not bound to the cellular cortex, is pulled by a SF. This demonstrates that a SF will strengthen faster as the ligand molecules become stiffer.

### Cell Detachment and Cell Rear Retraction

As SFs strengthened, FAs would disassemble less often due to the *FA*_*mat*_ mechanism. This increase in force was expected to rupture the rear FAs and cause the cell to retract its rear—a necessary step for migration. However, stabilization of FAs with force can also hinder migration, because not only are FAs in the rear stabilized, but also those in the front that are less likely to disassemble (due to the effect of parameter β in Equation 7). Disassembly of FAs in the front required an increase in rate of disassembly (*r*_*off, FA*_) with a drop in force after contraction—a mechanism implemented in accordance with observations that fiber strain induces disassembly of existing SFs (Sato et al., 2005). Thus, a factor, represented by φ, was added to Equation 9 to weaken fibers a certain amount of contraction, conducive of disassembly of the FA in the front.

The dependence of φ on the change in SF length (*L*_*fib*_) is described by a logistic function and presented in Figure 2C:

(10) $$\begin{array}{r} \varphi =\;\frac{1}{1+e^{-\mu \left(\Delta L_{fib}+\Delta L_{50}\right)}};\;\Delta L_{fib}=\;L_{fib}\left(t\right)-L_{fib}^{0} \end{array}$$

A small Δ*L*_*fib*_ leads to φ~1, thereby allowing contraction and promoting the strengthening of SFs. When *L*_*fib*_ has shortened by Δ*L*_50_, φ is halved (i.e., ${\vec{F}}_{SF,i}$ is divided by 2). Any further contraction would result in almost no force being applied by the SF, thereby promoting FA disassembly. μ describes the rate with which the force drops.

As Equation 5 indicates, SFs are not modeled as springs (common in other subcellular models), but rather the force drops only until a strain of approximately (−0.25, −0.4) is reached. Up until this point, the force acting on the nodes will pull them together until the SF is deleted when both FAs are disassembled or ruptured.

Even with this mechanism for SF weakening intended to destabilize FAs, a few FAs in the front Lm remained after retraction of the rear. These remaining FAs prevented the cell front from further extension. This is a limitation of the model, which is implemented in such a way that the mesh (representing the cellular surface) cannot slide past FAs or extend the cellular front by creating new plasma membrane at the cell front, as observed for cells *in vitro*. This limitation was overcome by disassembling all FAs after a full retraction of the rear occurs, followed by having all nodes in the Lm undergo a refractory period (λ_*ref*_); a full retraction event was said to occur when at least *n*_*thr*_ FAs ruptured within a span of 1 min. λ_*ref*_ provided time for extension of the cell front. The counter force (${\vec{F}}_{cnt}$) is particularly important at this point when all FAs are disassembled; it prevents the protrusion force (${\vec{F}}_{prot}$) from pulling the cell forward. The effects of this limitation are further discussed in the Discussion.

### Equation of Motion

Evolution of the system is described by the equation of motion. Because cells occupy a low Reynold's (overdamped) environment where inertial forces are negligible (Purcell, 1977), conservation of momentum for each node *i* takes the form:

(11) $$\begin{array}{l} \underset{conn.\;j}{\sum }{\vec{F}}_{linear,i}+{\vec{F}}_{A,i}+{\vec{F}}_{vol}+{\vec{F}}_{bend}+\underset{tri.\;l}{\sum }{\vec{F}}_{MD,i}+{\vec{F}}_{FA,i}\\ \;+{\vec{F}}_{SF,i}+\underset{tri.\;l}{\sum }{\vec{F}}_{prot,i}+\;{\vec{F}}_{cnt,i}\;\\ \;=\underset{conn.\;j}{\sum }\Lambda^{d}\left({\vec{v}}_{i}-{\vec{v}}_{j}\right)+\underset{tri.\;l}{\sum }\Gamma_{subs}{\vec{v}}_{i}+\;\Gamma_{liquid}{\vec{v}}_{i}\; \end{array}$$

The left-hand side contains the sum of all forces acting on each node at each time point. The linear force due to the springs in the Kelvin-Voigt model are summed for each node over all connections (conn.). As the Maugis-Dugdale and protrusion forces act on the triangles (tri.) in the mesh, forces are transfixed to the nodes (Odenthal et al., 2013).

The right-hand side shows the viscous force, described by the product of friction acting on node *i* (described by a friction tensor, Λ or Γ) and its velocity (${\vec{v}}_{i}$). The sources of friction are: dissipation of the actin cortex by all dampers *j* connected to node *i* (Λ^*d*^); contact with substrate triangles (Γ_*subs*_); and Stokes' drag (Γ_*liquid*_). The contributions of the friction to each node are added in a friction matrix at each time step, which together with the summation of forces can be used to solve for velocity and thus find the position in the next time step. A mathematical description of the dissipative forces (how friction tensors relate to friction coefficients) and how the friction matrix is built can be found in the Supplementary Material (section Dissipative Forces).

Equation 11 is a first-order differential equation that couples the movements of all nodes. More information on the numerical solution of the system can be found in the Supplementary Material (section Numerical Solution and Implementation). A time step of 0.05 s was used in all simulations. We chose the largest timestep possible that allowed the simulation to run smoothly (i.e., without numerical instabilities). There is a trade-off between accuracy and computational efficiency when it comes to timestep duration: A larger timestep (i.e., one order of magnitude larger) would result in a larger displacement of nodes per timestep leading to fluctuations, or worse, the cell nodes moving across surfaces (e.g., substrate triangles). A shorter timestep would have provided the same results and reduce the risk of any instability; however, it makes the simulation slower computationally. All simulations were performed using the C++ particle-based software Mpacts (http://mpacts.com).

At the time of publication, Mpacts is a closed-source software. To ensure reproducibility, however, we made use of Docker platform (www.docker.com). Docker allows recreation of an exact runtime computational environment. With the reproducibility package, available in (https://gitlab.kuleuven.be/MAtrix/mpact-docker-reproduce-cellmig.git), one can create a runtime environment similar to that we used to run simulations presented in this work. This package allows creation of a ready-to-be-used Linux machine with Mpacts software and its dependencies installed in it. The user may change the mechanosensing setup, the parameter values in the simulation script, run a cell migration simulation through Docker, and visualize the results using visualization software, such as Paraview (www.paraview.org). Documentation for all commands, including those used in the simulation script provided can be found in http://dev.mpacts.com/documentation/index.html. A detailed description of how to run the reproducible simulation and a description of all provided files can be found in GitLab.

### Parameter Study Design

We varied substrate stiffness (*k*_*ECM*_) and FA disassembly rate ($r_{off,FA}^{0}$, representative of varying degree of adhesion receptor-ligand affinity) for four *setups*, defined by all possible combinations of the two mechanosensing mechanisms implemented in the model (i.e., *FA*_*mat*_ and *SF*_*str*_) being either ON or OFF. The distinct values of *k*_*ECM*_ and $r_{off,FA}^{0}$ used are presented in Table 2; we refer to each pair of *k*_*ECM*_ and $r_{off,FA}^{0}$ values used as defining a *condition*.

**Table 2:** Parameter values for study (***k***_***ECM***_ and $r_{off,FA}^{0}$) and corresponding approximations (***E***_***ECM***_ and ${\langle \lambda \rangle }_{FA}^{0}$).

**Parameter**	**Values**	**Units**
***K*_*ECM*_**	[1 × 10^−3^, 3.5 × 10^−3^, 1.2 × 10^−2^, 4.16 × 10^−2^, 1.44 × 10^−1^, 5 × 10^−1^]	N/m
***E*_*ECM*_**	[0.25, 0.87, 3, 10.4, 36, 125]	kPa
$r_{off,FA}^{0}$	[5 × 10^−2^, 1.08 × 10^−2^, 2.3 × 10^−3^, 5 × 10^−4^]	1/s
${\langle \lambda \rangle }_{FA}^{0}$	[0.3, 1.5, 7.2, 33.3]	min

The values of *k*_*ECM*_, corresponding to spring stiffness, can be converted into approximate Young's moduli found in Table 2 (see Supplementary Material for conversion, section Discrete Adhesions and Substrate Stiffness) (Mitrossilis et al., 2009). When converted, the range of substrate stiffness values considered corresponds to (0.25,125) kPa. This is the range over which certain cell types have shown their entire range of cell speed—for example, U87 glioma cells displaying mesenchymal migration (Ulrich et al., 2009) and smooth muscle cells (Peyton and Putnam, 2005).

For each value of $r_{off,FA}^{0}$, the corresponding expected FA lifetime value (under no force) of ${\langle \lambda \rangle }_{FA}^{0}$ is provided according to Equation 8. These values will be used to display results because a lifetime is more intuitive than a rate. The disassembly rate ($r_{off,FA}^{0}$) was in accordance with studies showing that affinity in the form of disassembly rate can be altered by altering the ligand (Müller and Pompe, 2016). Simulated FAs, even when stabilized by force (i.e., from *FA*_*mat*_), were found to live up to 400 min but on average up to 100 min; thus, the values of $r_{off,FA}^{0}$ chosen produce FAs that have lifetimes in the order of magnitude of FAs studied experimentally: (20−200) min (Berginski et al., 2011).

By combining the parameter values, 24 different conditions were simulated for each setup. Each condition was simulated five times to be able to average output to quantify the effect of stochasticity in the model stemming from the probabilistic nature of FA formation and disassembly.

### Output Metric Calculation

To analyze the results, six metrics were extensively studied: the cell displacement, the actual lifetime of FAs, the strengthening factor of the SFs (*n*_*str, f*_), the number of FAs, the number of ruptured FAs (FAs that disassembled when $|{\vec{F}}_{FA}|\geq F_{rup}$), and the traction exerted by the cell on the substrate. All of the aforementioned metrics at each recorded time point are direct outputs of the model, with the exception of displacement and traction, which require further calculations.

Displacement is calculated from the position of the center of mass (CoM) of the nodes in the cell–substrate interface. It corresponds to the change in *x* position of the CoM between consecutive time points and summing up their values for the entire simulation. To calculate cellular traction, the traction exerted by the cell on each substrate triangle *i* (${\vec{T}}_{\Delta i}$) was first calculated according to:

(12) $$\begin{array}{r} {\vec{T}}_{\Delta i}=\frac{1}{A_{\Delta }}\sum_{j=\;0}^{n_{FA,\Delta }}{\vec{F}}_{FA,j} \end{array}$$

where ${\vec{F}}_{FA,j}$ is the force exerted by FA *j*, *A*_Δ_ is the area of a substrate triangle, and *n*_*FA*, Δ_ is the number of FAs bound to the triangle. A sample traction map showing the traction magnitude values per triangle is presented in the (Figure S2D).

Total cell traction magnitude (${|\vec{T}}_{cell}|$) follows by summing up the traction magnitude over all substrate component triangles (*n*_Δ, *subs*_):

(13) $$\begin{array}{r} {|\vec{T}}_{cell}|=\sum_{j=0}^{n_{\Delta ,subs}}|{\vec{T}}_{\Delta i}| \end{array}$$

## Results

Traditionally, retraction of the rear has been seen as triggered by rupture of FAs (Chen, 1981). This rupture has been associated with recoiling of pseudopodia with cytoskeletal contraction. While some cells visually contract and display a pause between displacement steps (e.g., epithelial cells, fibroblasts, and cancer cells) (Sahai and Marshall, 2003; Friedl and Wolf, 2010; Chang et al., 2013), other cell display smooth migration with little change in cell shape in time (e.g., keratocytes) (Lee et al., 1993). Rather than assuming rupture occurs, some models attribute retraction to an imbalance in stability of FAs in the front and rear (Kim et al., 2013).

**Figure 3:**
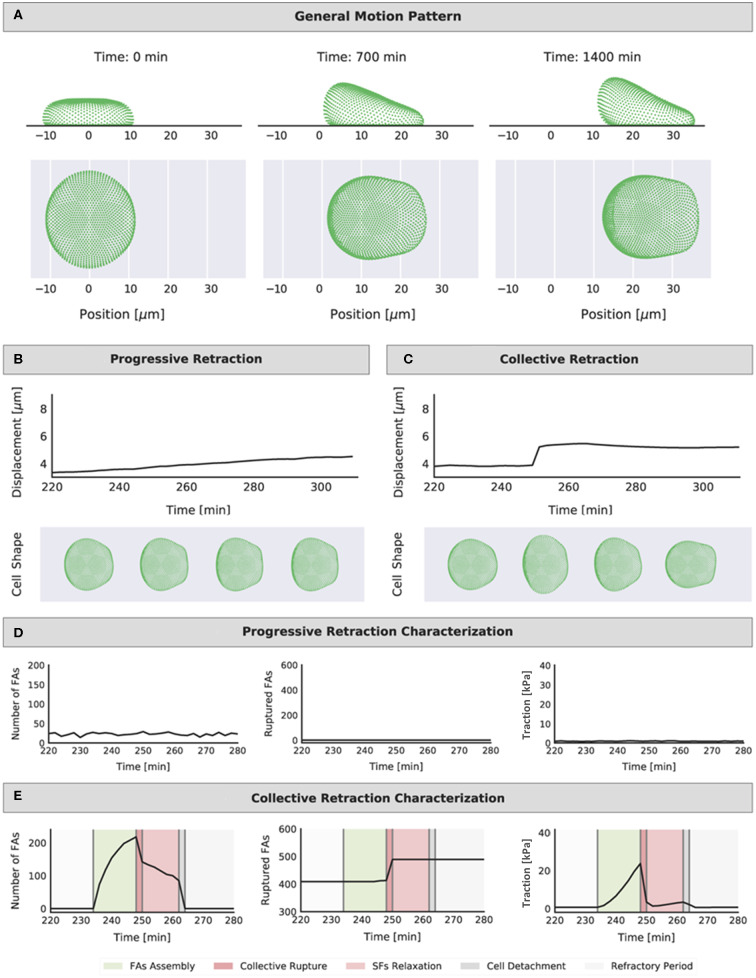
Representation of the position of the cell cortex (side and top view) at different time points (start, middle, and end of simulation) **(A)**. Example representations of the cell displacement and evolution of shape during a 60-min interval for the two identified migration modes: **(B)** progressive retraction (mechanosensing setup: *FA*_*mat*_[*ON*] || *SF*_*str*_[*ON*], condition: *k*_*ECM*_ = 0.0416 N/m, ${\langle \lambda \rangle }_{FA}^{0}=0.3$ min) and **(C)** collective retraction (mechanosensing setup: *FA*_*mat*_[*ON*] || *SF*_*str*_[*ON*], condition: *k*_*ECM*_ = 0.5 N/m, ${\langle \lambda \rangle }_{FA}^{0}=33.3$ min). Shape shown for following intervals: *t* = 230 min (FA assembly), *t* = 250 min (collective rupture), *t* = 260 min (cell detachment), and *t* = 280 min (refractory period). Representations of the number of FAs at each interval (not cumulative), the cumulative number of ruptured FAs, and the traction values in time for corresponding simulations: **(D)** progressive retraction and **(E)** collective retraction (shading highlights the different events in a force build-up and rupture part of the migration cycle). The same time interval of 60-min was chosen for display to compare quantities in both retraction modes; this is the approximate duration of a single iteration of the migration cycle. The range in the *y* axes is also the same for each quantity in each mode for easier comparison.

We noticed that for the different setups and parameter values (i.e., *k*_*ECM*_ and ${\langle \lambda \rangle }_{FA}^{0}$) simulated cells displayed either smooth or cyclical migration. We tracked displacement, number of FAs over time as well as rupture events due to excessive force, and cellular traction (see section Output metric calculation in Methods): Two distinct migration modes arose characterized by either a progressive or collective retraction, corresponding to smooth and cyclical migration, respectively. Both modes allowed cells to migrate, yet they occurred in distinct areas of the parameter space defined by the values selected for *k*_*ECM*_ and ${\langle \lambda \rangle }_{FA}^{0}$. The incidence was also affected by the mechanosensing mechanisms implemented. Snapshots of a migrating cell and a summary of the differences between migration modes are presented in Figure 3.

Cells displaying progressive retraction had a smooth displacement curve in time. Cell shape did not vary significantly in time, and the number of FAs at any given instant and traction levels were low (Figures 3B,D). There were very few or no ruptured FAs; therefore, there was no need for detachment of the entire cell for migration to occur. At each time point the assembly and disassembly of FAs were more or less balanced, leading to FA numbers that did not fluctuate much in time (Figure 3D); hence, these cells can be thought of as low-adhered cells that constantly moved forward without changing its shape, similar to keratocytes (Lee et al., 1993). Migration occurred as SFs, connecting a pair of nodes (one in the front and one in the rear), would pull forward the node in the rear once the FA disassembled, shifting incrementally the position of the cell in the forward direction. Meanwhile, protrusion force in the front Lm ensured that detached nodes in the front would themselves shift forward, such that they can potentially pull other nodes in the rear if connected by a SF. An animation of a migrating cell displaying progressive retraction is presented in Videos S1–S6, which show in time the evolution of the traction map on the substrate, shape of front and rear lamella, FAs, time elapsed since rear FA detachment for each SF, strengthening factor (*n*_*str, f*_) per SF, and nodes in a refractory state (captions and links to videos are found in the Supplementary Material).

Cells displaying collective retraction underwent collective rupture of FAs at the rear with every displacement step; collective rupture occurred when the force threshold (*F*_*rup*_) was reached for multiple FAs in the rear, causing a sudden retraction event. This displacement of nodes in the rear was much larger than that caused by contraction of individual fibers in the progressive retraction mode. As such, the displacement curve showed step-like behavior, indicating that, rather than a continuous motion, the cell stalled for some minutes in each step. Contrary to what was seen for progressive retraction, there was no balance between assembly and disassembly of FAs. Instead, there was a cyclic process where assembly dominated until rupture would occur. The cell was then forced to detach and entered a refractory period, restarting the cycle (see also shaded areas in Figure 3D). An animation of a migrating cell displaying collective retraction is presented in Videos S7–S12, which show in time the evolution of the traction map on the substrate, shape of front and rear lamella, FAs, time elapsed since rear FA detachment for each SF, strengthening factor (*n*_*str, f*_) per SF, and nodes in a refractory state (captions and links to videos are found in the Supplementary Material).

Collective rupture at the rear is explained because the cortex was viscoelastic; force applied by SFs could be carried by the cortex to neighboring nodes, leading to a total force on a node that is higher than that applied by single SFs on individual FAs. As more FAs ruptured (and with corresponding SFs still applying force as long as they remained bound at a FA in the front), there was a compounding effect and all FAs in the rear ruptured. This event is comparable to the collective failure of adhesion molecules (or clutches) modeled by Chan and Odde at a single point in space, corresponding to an adhesion cluster (Chan and Odde, 2008). In our model, collective failure occurs over a large 2D section of the cell-substrate interface (i.e., the entire rear Lm) and is mediated by cortex stiffness.

**Figure 4:**
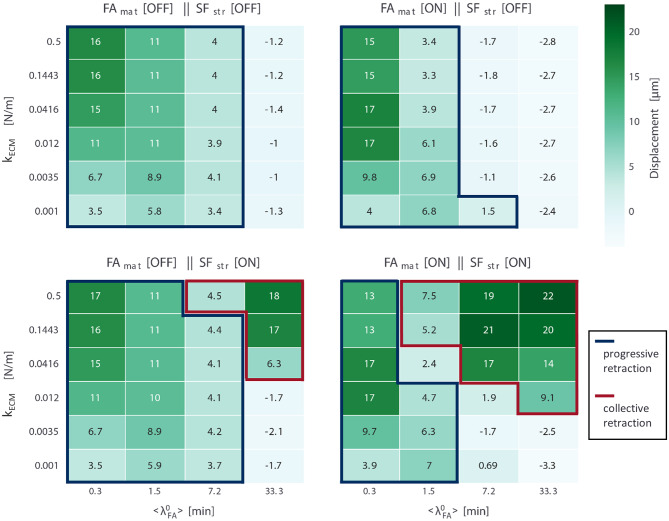
Average displacement values (*N* = 5) for simulations for all four mechanosensing setups (*FA*_*mat*_ and *SF*_*str*_ being either ON or OFF); for each setup, 24 conditions were run, defined by parameter values for substrate stiffness (*k*_*ECM*_, six values) and expected FA lifetime under no force (${\langle \lambda \rangle }_{FA}^{0}$, four values). Cells in conditions encased by blue lines displayed progressive retraction, while those encased by red lines displayed collective retraction. Values correspond to displacements over the entire duration of the simulations (24 h).

The transition between progressive and collective retraction modes with changing parameter values provides insight into cell migration. Average displacement values at the end of the simulations for all conditions and all setups are presented in Figure 4; each of these displacement maps can be interpreted as a sort of phase diagram describing what migration modes a cell adopts under different ECM conditions (defined in terms of substrate stiffness and adhesion receptor–ligand affinity), given the mechanosensing mechanisms present subcellularly. Conditions are marked as areas of progressive retraction if, regardless of speed, cells continuously moved forward throughout the entire simulation, while conditions are marked as areas of collective retraction if a full retraction event occurred throughout the simulation. Otherwise, we consider that there is no migration in a condition. Thus, a careful look at the differences in these displacement maps provide insight into the role of each mechanosensing mechanism (*FA*_*mat*_ and *SF*_*str*_) on migration and their sensitivity to substrate stiffness and adhesion lifetime. A negative displacement value corresponds to no displacement; contraction of the cell body without migration shifts the center of mass in the negative direction yielding a negative value due to unhindered movement of nodes not in contact with the substrate, due to force counter to protrusion (${\vec{F}}_{cnt}$). Thus, conditions not classified as displaying either progressive or collective migration either only shifted their CoM in the negative *x* direction by elongating or contracted shifting the CoM in the positive *x* direction by <3 μm early in the simulation, but did not manage to trigger a full rupture event and remained attached to the substrate. The average displacement (and standard deviation) per setup over all conditions in which cells migrated were: *FA*_*mat*_[*OFF*] || *SF*_*str*_[*OFF*] 8.35 ± 4.53 μ*m*, *FA*_*mat*_[*ON*] || *SF*_*str*_[*OFF*] 8.44 ± 5.67 μ*m*, *FA*_*mat*_[*OFF*] || *SF*_*str*_[*ON*] 9.2 ± 5.02 μ*m*, and *FA*_*mat*_[*ON*] || *SF*_*str*_[*ON*] 11.27 ± 7.02 μ*m*.

For another depiction of the same data (cell displacement values for all setups and conditions), check Figure S7. Rather than in a heat map, average displacement values per condition are presented in scatter plots for each adhesion receptor-ligand affinity value ($\langle \lambda_{FA}^{0}\rangle$). This presentation shows dependence on substrate stiffness very clearly and shows variability between replicate simulations by plotting standard error of the mean. The same style plots are presented for the other metrics: actual lifetime of FAs (Figure S8), the strengthening factor of the SFs (*n*_*str, f*_) (Figure S9), the number of FAs (Figure S10), and the cumulative number of ruptured FAs (FAs that disassembled when $|{\vec{F}}_{FA}|\geq F_{rup}$) (Figure S11).

## Discussion

The simulated cells were much slower than what is seen *in vitro*. For the condition in which cells migrated the most (22 μm) in the 24 h simulated period (*FA*_*mat*_[*ON*] || *SF*_*str*_[*ON*] with *k*_*ECM*_= 0.5 N/m, ${\langle \lambda \rangle }_{FA}^{0}=$ 33.3 min), the corresponding speed is 0.015 μm/min. In literature, slow mesenchymal cells have been characterized by speed values of 0.1–0.5 μm/min (Friedl et al., 1998; Wu et al., 2013).

The slow speed of the simulated cells, regardless of condition, can be attributed mainly to the inability of the cell to protrude much beyond the FAs that are closest to the front edge of the cell, which comes from the use of a mesh with immutable connections between nodes throughout the entirety of simulations. In reality, cells are able to disassemble their cortex and extend the fluid-like membrane well-beyond their point of adhesion (Giannone et al., 2007). The current shortcoming of the model was addressed with a detachment step after retraction followed by a refractory period that allows the front to extend (section Cell detachment and cell rear retraction). This period made each step longer, making the simulated cells slower. Regardless, retraction is a mechanosensitive process in the model and the comparison between conditions is nevertheless insightful. Additionally, the speed of retraction of the rear was fast (approximately 16 μm/h). Thus a larger cell with a realistic radius (e.g., 25–50 μm) would already be significantly faster since each contraction step would already move the cell body more, yet this would be more computationally expensive to simulate.

### When Fibers Cannot Mature, Excessive Adhesion Hinders Migration

**Figure 5:**
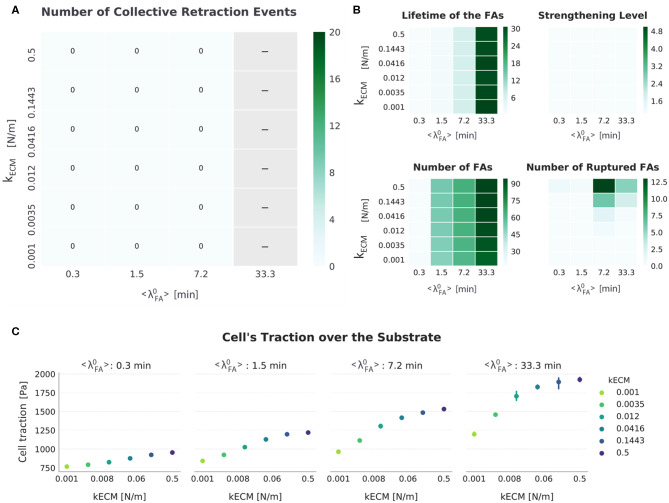
Summarized results for *FA*_*mat*_[*OFF*] || *SF*_*str*_[*OFF*]. **(A)** The top-left heat map presents the number of collective retraction events per condition; gray corresponds to conditions with no displacement. There was no collective rupture for this condition, and all cells displayed progressive retraction. **(B)** Other heat maps (top right) show the average actual lifetime of the FAs, the strengthening level of the SFs (*n*_*str, f*_), the average number of FAs, and the cumulative number of ruptured FAs. **(C)** The scatter plots (bottom) show average traction over the last hour of simulation. Error bars correspond to standard error of the mean (*N* = 5).

For the setup *FA*_*mat*_[*OFF*] || *SF*_*str*_[*OFF*], all migrating cells displayed progressive retraction. From the displacement values (Figure 4), it can be concluded that cells migrated more on stiffer substrates. This effect was only apparent for lower expected FA lifetimes (${\langle \lambda \rangle }_{FA}^{0}$). Detailed presentation of the results obtained for metrics used to analyze the simulations for this setup are presented in Figure 5. To compare across conditions, a single representative value of each output metric was calculated and reported for time varying values. Since both the displacement and the number of ruptured FAs are cumulative quantities, their representative value is the value at the end of the simulation. For the remaining metrics (i.e., actual lifetime of FAs, strengthening factor *n*_*str, f*_, number of FAs, and traction exerted by the cell ${|\vec{T}}_{cell}|$), as these are average values of the existing FAs and SFs at a certain time point, the representative value is computed for a particular time interval in the migration step where the value is stable: For cells that migrate via progressive retraction, this interval corresponds to the last 800 min of the simulation. For cells that undergo collective retraction and detach for a refractory period (λ_*ref*_), the values are stable immediately before detachment; the representative value is thus given by the average of values for a 10 min interval, immediately before all the detachment events the cell undergoes.

In the absence of *SF*_*str*_, the only difference in average lifetime of FAs is introduced by ${\langle \lambda \rangle }_{FA}^{0}$. Cells migrated when FA assembly and disassembly are in balance, which occur for relatively low ${\langle \lambda \rangle }_{FA}^{0}$ values. For high ${\langle \lambda \rangle }_{FA}^{0}$ values, FA assembly occurs more often than disassembly, resulting in cells too attached to migrate, as suggested in experimental studies (Nagano et al., 2012). There was only significant migration for cells with a high turnover rate of FAs (${\langle \lambda \rangle }_{FA}^{0}\leq$ 1.5 min). In contrast, for lifetime values closer to those recorded *in vitro* (20–40 min) (Berginski et al., 2011; Stehbens and Wittmann, 2014), the number of FAs is higher, thereby hindering migration. Thus, without *FA*_*mat*_ and *SF*_*str*_, a mesenchymal cell with a FA lifetime between 20 and 40 min (as seen experimentally) would not migrate.

Regarding the role of *k*_*ECM*_, there was a small increase in number of FAs with increasing *k*_*ECM*_ (Figure 5 and Figure S10). This is explained by the limited cell deformation that occurs on stiffer substrates relative to cells on softer substrates, where the springs representing ligand molecules could be further deformed; the limited deformation results in a larger Lm in which a few more FAs could be formed. This difference in size of cell–substrate interface due to different substrate stiffness has been observed experimentally: When cells are able to deform the substrate, they adopt a more round, less spread state (Wells, 2008; Polio et al., 2019). Regarding the number of ruptured FAs in this setup, the values are very low compared to other setups, with a maximum average of 12.5 FAs rupturing due to force in any condition during a simulation. More FAs ruptured in conditions corresponding to a high substrate stiffness relative to conditions with low substrate stiffness (Figure S11). This can be explained from the fact that even without *SF*_*str*_, force values on FAs bound to SFs rise faster on stiff substrates (see first step in Figure 2B), and for longer-lived FAs that would lead to more SFs exerting force.

Furthermore, there was an increase in traction with increasing *k*_*ECM*_, which can be attributed to both the increased number of FAs and limited weakening of fibers upon shortening (Figure 2C) due to smaller strain of stiffer springs representing the ligand. Hence, for this setup (*FA*_*mat*_[*OFF*] || *SF*_*str*_[*OFF*]), higher tractions are associated with more migration, but only in cells with low adhesion to the substrate. This is comparable to behavior described in the literature for epithelial cells (Onochie et al., 2019). These results suggest that cell traction is a function of both *k*_*ECM*_ and ${\langle \lambda \rangle }_{FA}^{0}$, where the effect of each one on migration is difficult to quantify. Therefore, traction by itself is not sufficient to predict the displacement of the cell, but understanding the causes behind the differences in traction is insightful.

### Strengthening of SFs Enables Collective Retraction

**Figure 6:**
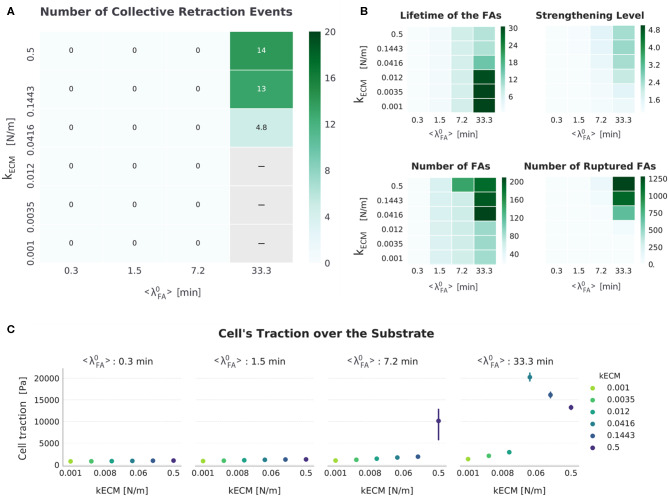
Summarized results for *FA*_*mat*_[*OFF*] || *SF*_*str*_[*ON*]. **(A)** The top left heat map presents the number of collective retraction events per condition; gray corresponds to conditions with no displacement. Collective rupture for this condition required a stiff substrate and long-lived FAs. **(B)** Other heat maps (top right) show the average actual lifetime of the FAs, the strengthening level of the SFs (*n*_*str, f*_), the average number of FAs, and the cumulative number of ruptured FAs. **(C)** The scatter plots (bottom) show average traction over the last hour of simulation. Error bars correspond to standard error of the mean (*N* = 5).

For the setup *FA*_*mat*_[*OFF*] || *SF*_*str*_[*ON*], cells displayed both progressive and collective retraction. For conditions corresponding to short-lived FAs (${\langle \lambda \rangle }_{FA}^{0}\;\leq$ 7.2 min), retraction was progressive and displacement values were very similar to the case when no mechanosensing mechanism was active (*FA*_*mat*_[*OFF*] || *SF*_*str*_[*OFF*]) (Figure 4). Yet, there was a second area in the parameter space where equally high displacements occurred and retraction was collective and that was characterized by high substrate stiffness (*k*_*ECM*_ ≥ 0.0416 N/m) and long-lived adhesions (${\langle \lambda \rangle }_{FA}^{0}=$ 33.3 min). The use of collective retraction by cells in these conditions is evidenced by high rupture levels and resulting decrease in the average lifetime of FAs (Figure 6).

Considering how *SF*_*str*_ was implemented (section SF formation, contraction, and strengthening), the results here indicate that, for short-lived adhesions (${\langle \lambda \rangle }_{FA}^{0}\;\leq$ 7.2 min), SFs were able to keep contracting without stalling, preventing them from strengthening (see Figure 6, top right heat map). This matches observations that SFs are usually not found in cells with short-lived adhesions despite presenting contractile actin structures (Lämmermann and Sixt, 2009).

In the parameter space where collective retraction was observed (outlined in Figure 4), Figure 6 shows how an increase in *k*_*ECM*_ resulted in more collective retraction events (implying they also occurred with a higher frequency), which also explains an increase in the number of ruptured FAs (Figure S11), a decrease in number of FAs (Figure S10), and a decrease in average lifetime of the FAs (Figure S8). This is also why there was a decrease in traction with increasing stiffness in this same region; although this has been observed experimentally, it was associated with a corresponding decrease in cellular speed (Chan and Odde, 2008)—not the opposite as shown here. As substrate stiffness increased, the increase in FA rupture (Figure 6A) results in less traction is exerted. This meant the cell was less attached and thus could migrate faster.

In direct contrast with the setup in which no mechanosensing mechanisms is active (*FA*_*mat*_[*OFF*] || *SF*_*str*_[*OFF*]), a high receptor-ligand affinity (${\langle \lambda \rangle }_{FA}^{0}$ = 33.3 min) is needed for cells to migrate. Combined with high substrate stiffness, both factors cause SFs to mature enough to enable FA rupture and collective retraction (see *n*_*str, f*_ values, Figure 6). Furthermore, *k*_*ECM*_ plays an important role in determining the rate at which retraction events occur.

As discussed previously, this cyclic collective rupture to which migration can be attributed can be compared to “load-and-fail" events proposed in the clutch model of adhesion clusters, where high values of force are built and ultimately lead to the rupture of multiple adhesions (Bangasser et al., 2013). The clutch model uses a catch-slip bond formulation of adhesions, where adhesions strengthen with force with initial force application (catch part), but then weaken with a further increase in force (slip part). In this case, in which a threshold force elicits rupture (*F*_*rup*_), the FAs act as slip bonds. Decrease in traction can be similarly attributed to increased adhesion rupture and was observed in all setups in which collective retraction occurred (whether a catch component in the form of *FA*_*mat*_ was present or not).

To further illustrate the effect introduced by the activation of *SF*_*str*_, we calculated the difference between displacement and other metrics for the setups thus far discussed (i.e., *FA*_*mat*_[*OFF*] || *SF*_*str*_[*OFF*] and *FA*_*mat*_[*OFF*] || *SF*_*str*_[*ON*]). This is presented as Figure S12.

### FA Maturation Without SF Strengthening Further Inhibits Cell Migration

**Figure 7:**
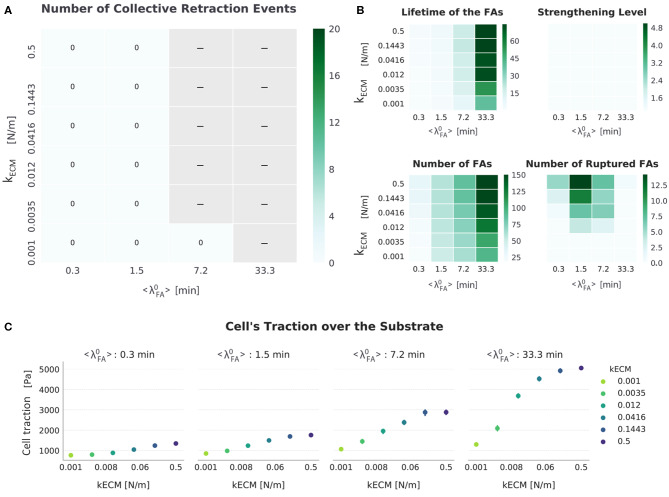
Summarized results for *FA*_*mat*_[*ON*] || *SF*_*str*_[*OFF*]. **(A)** The top-left heat map presents the number of collective retraction events per condition; gray corresponds to conditions with no displacement. There was no collective rupture for this condition, and all cells displayed progressive retraction. **(B)** Other heat maps (top right) show the average actual lifetime of the FAs, the strengthening level of the SFs (*n*_*str, f*_), the average number of FAs, and the cumulative number of ruptured FAs. **(C)** The scatter plots (bottom) show average traction over the last hour of simulation. Error bars correspond to standard error of the mean (*N* = 5).

For the setup *FA*_*mat*_[*ON*] || *SF*_*str*_[*OFF*], all migrating cells displayed progressive retraction. Displacements in each condition, defined by *k*_*ECM*_ and ${\langle \lambda \rangle }_{FA}^{0}$, were similar to the corresponding condition in the setup where no mechanosensing mechanism was active (*FA*_*mat*_[*OFF*] || *SF*_*str*_[*OFF*]) (Figure 4). A detailed presentation of metrics used to analyze the simulations for this setup are presented in Figure 7.

As in the setup *FA*_*mat*_[*OFF*] || *SF*_*str*_[*OFF*], both increasing *k*_*ECM*_ and ${\langle \lambda \rangle }_{FA}^{0}$ promote a spread-adhered morphology, reflected by an increase in the number of FAs and cell tractions (Figure 7). Implementing the additional *FA*_*mat*_ mechanism increased the actual lifetime of FAs. The inhibition of migration by excessive adhesion occurred in this setup as well, but starting in conditions with a lower value of ${\langle \lambda \rangle }_{FA}^{0}$: Whereas in *FA*_*mat*_[*OFF*] || *SF*_*str*_[*OFF*] simulations migration stalled when ${\langle \lambda \rangle }_{FA}^{0}$ = 33.3 min, in *FA*_*mat*_[*ON*] || *SF*_*str*_[*OFF*] simulations migration stalls when at ${\langle \lambda \rangle }_{FA}^{0}\;\geq$ 7.2 min.

In contrast to results observed for the setup *FA*_*mat*_[*OFF*] || *SF*_*str*_[*OFF*], an increase in *k*_*ECM*_ resulted in decreased cell displacement (Figure 4). This occurred because the longer lifetime of FAs gave SFs time to stall (*F*_*ECM*_ = *F*_*am*_); this occurs sooner the stiffer the substrate is (see first step increase in Figure 2B). There was also a compounding effect where the increase in FAs due to longer lifetimes also results in more SFs, which in turn further increase the force exerted by the cells, thereby stabilizing adhesions.

It is worth mentioning that the trend in number of ruptured FAs also seems to have shifted to conditions with a lower ${\langle \lambda \rangle }_{FA}^{0}$ (compared to the setup *FA*_*mat*_[*OFF*] || *SF*_*str*_[*OFF*]). This meant that as ${\langle \lambda \rangle }_{FA}^{0}$ increased, there was a peak in number of ruptured FAs at intermediate values of ${\langle \lambda \rangle }_{FA}^{0}$. This was surprising, because, as mentioned, longer living adhesions always resulted in higher cellular traction. Although there was more force being exerted for higher values of ${\langle \lambda \rangle }_{FA}^{0}$, this force was distributed through more FAs, eventually making the force a single FA withstood actually lower such that it did not reach the rupture force. Not enough force was built up to cause a collective rupture event.

### When Both Mechanosensing Mechanisms Are Included, Migration via Collective Retraction Occurs With Parameters Values That Match Physiological Values

**Figure 8:**
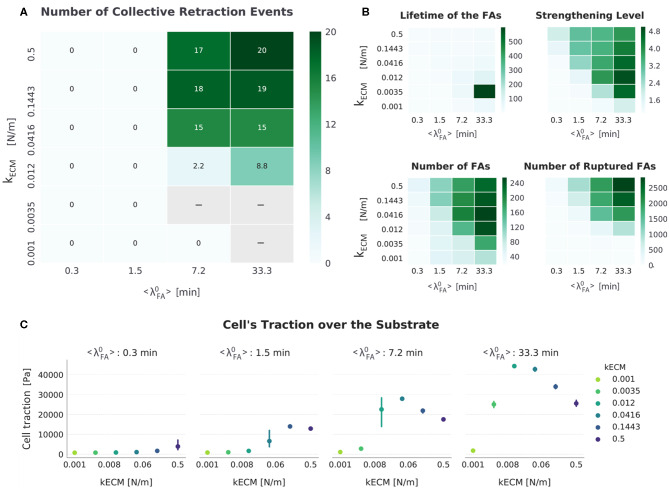
Summarized results for *FA*_*mat*_[*ON*] || *SF*_*str*_[*ON*]. **(A)** The top-left heat map presents the number of collective retraction events per condition; gray corresponds to conditions with no displacement. Collective rupture occurred over a larger range than any other setup and displayed a bimodal relation between cellular traction and substrate stiffness (*k*_*ECM*_). **(B)** Other heat maps (top right) show the average actual lifetime of the FAs, the strengthening level of the SFs (*n*_*str, f*_), the average number of FAs, and the cumulative number of ruptured FAs. **(C)** The scatter plots (bottom) show average traction over the last hour of simulation. Error bars correspond to standard error of the mean (*N* = 5).

Analyzing the results when both mechanisms are implemented (*FA*_*mat*_[*ON*] || *SF*_*str*_[*ON*]), Figure 8 shows that the effect of each mechanism independently can be observed. In the case of *FA*_*mat*_, progressive migration is inhibited in conditions with a relatively high ${\langle \lambda \rangle }_{FA}^{0}$ (${\langle \lambda \rangle }_{FA}^{0}=$ 7.2 min) and shifts to conditions with lower values (${\langle \lambda \rangle }_{FA}^{0}\leq$ 1.5 min). *SF*_*str*_, instead, was responsible for migration through collective retraction at high ${\langle \lambda \rangle }_{FA}^{0}$ values (${\langle \lambda \rangle }_{FA}^{0}\geq$ 7.2 min). The parameter space became dominated by collective retraction. This joint implementation of mechanosensing mechanisms resulted in the highest displacement values observed overall for any setup, with an average displacement of 11.27 ± 7.02 μm over all conditions in which cells migrated (Figure 4).

This setup (*FA*_*mat*_[*ON*] || *SF*_*str*_[*ON*]), however, was not the one for which cells migrated in most conditions (as defined by combination of parameters *k*_*ECM*_ and ${\langle \lambda \rangle }_{FA}^{0}$); it was rather the setup *FA*_*mat*_[*OFF*] || *SF*_*str*_[*ON*] (Figure 4). Both mechanosensing mechanisms needed to be active though to ensure migration via collective retraction for cells with low receptor-ligand affinity (${\langle \lambda \rangle }_{FA}^{0}=$ 7.2 min). This better simulates actual cells, for which most FAs are short-lived, unless reinforced by cytoskeletal forces to reach average lifetimes of 20 to 40 min.

This setup also presents a clear subset of conditions (extended in comparison to other setups) for which there is no migration: low substrate stiffness (*k*_*ECM*_ ≤ 3.5 × 10^−3^ N/m) and high receptor-ligand affinity (${\langle \lambda \rangle }_{FA}^{0}\geq$ 7.2 min). A gap signifying no cell migration between parameter regions for the two modes widened relative to the other setup in which both migration modes were observed (*FA*_*mat*_[*OFF*] || *SF*_*str*_[*ON*]) (Figure 4). In these conditions, the cell would occasionally retract its rear collectively, but mostly it was too attached to migrate progressively and too weak to rupture multiple FAs in the rear.

### When Both Mechanosensing Mechanisms Are Included, Bimodal Dependence of Traction on Substrate Stiffness Ensues

Another emergent property of the system is the bimodal dependence of cellular tractions on substrate stiffness, observed when ${\langle \lambda \rangle }_{FA}^{0}\geq$ 1.5 min (Figure 8). This meant that for a certain range of *k*_*ECM*_ values, cell traction decreased with increasing stiffness. An increase in cellular traction was observed for the *k*_*ECM*_ range (0.25,3) kPa; this behavior was observed for the same range in Human Umbilical Vein Endothelial Cells (HUVECs) (Izquierdo-Álvarez et al., 2018). In this study, HUVECs were shown to exert a total force between 0.1 and 0.45 μN. Migrating simulated cells in the setup *FA*_*mat*_[*ON*] || *SF*_*str*_[*ON*] exerted a total force between 0.036 and 0.084 μN. This means simulated cells were 3–6 × weaker, yet smaller in area (~40 ×).

For conditions with the highest receptor–ligand affinity in which cells migrated (${\langle \lambda \rangle }_{FA}^{0}=33.3$ min and *k*_*ECM*_ (0.012,0.5) N/m, Figure 4), as displacement (i.e., speed) increased, traction force decreased (see scatter plot Figure 8). This means cells were the most efficient on the stiffest substrates when having the highest receptor-ligand affinity. In these conditions with high affinity, cells would slow down on soft substrates because not enough FAs ruptured—a fact attributed to both a high strengthening level (*n*_*str, f*_) and resulting high FA lifetime due to *FA*_*mat*_. One particular condition (*k*_*ECM*_ = 0.0035 N/m and ${\langle \lambda \rangle }_{FA}^{0}$ = 33.3 min) stood out in that the cell would often become stuck, evidenced by the high FA lifetime (~600 min) and very low number of retraction events (8.8). This condition presented the highest tractions of all simulations.

To further illustrate the effect introduced by the activation of *SF*_*str*_ when there is *FA*_*mat*_, we calculated the difference between displacement and other metrics for setups *FA*_*mat*_[*ON*] || *SF*_*str*_[*OFF*] and *FA*_*mat*_[*ON*] || *SF*_*str*_[*ON*]). This is presented as Figure S13.

## Conclusions

In agreement with experimental observations, two distinct migration modes were observed in simulations, distinguishable through the way in which the rear of the cell is retracted: progressive retraction vs. collective retraction. It is known that distinct cell types migrate differently and that the environment can trigger changes in cell migration mode. Our model shows that two substrate-associated properties—specifically, substrate stiffness and adhesion receptor-ligand affinity—cause cells to adopt one of two migration modes.

Novel in its implementation of two mechanosensing mechanisms, *FA*_*mat*_ and *SF*_*str*_, the model uncovers the individual role of these subcellular processes in cell migration. *FA*_*mat*_ is responsible for hindrance of migration on stiff substrates. Meanwhile, *SF*_*str*_ enables collective retraction. Both mechanisms, however, were found to be necessary to enable mesenchymal migration in a realistic range of substrate stiffness. With realistic force magnitudes applied per FA, the model also generated traction maps that showed spatiotemporal traction distributions that agree with experimentally generated traction maps (see Videos in Supplementary Material).

Despite limitations in the model, these findings are useful to interpret experimental results of studies in which substrate stiffness and adhesion ligand itself are varied; changing the ligand molecule entails a change in adhesion receptor–ligand affinity. The simulated results also provide a guide for experimental design, in which a phase diagram defined by substrate-associated properties can be used to elicit an initial target migration from cells. In addition, the model provides a theoretical tool to study how changes to other molecular components of focal adhesions and cytoskeleton may affect mechanosensing and migration.

## Data Availability Statement

The datasets generated for this study are available on request to the corresponding author.

## Author Contributions

DV, HR, and HV designed the study. DV, TH, and BS conceived the model. DV implemented the model. DV, IG, and LL-G ran simulations and analyzed results. DV and IG wrote the article.

## Conflict of Interest

The authors declare that the research was conducted in the absence of any commercial or financial relationships that could be construed as a potential conflict of interest.
