# Supplementary material for: Modeling of Mechanosensing Mechanisms Reveals Distinct Cell Migration Modes to Emerge From Combinations of Substrate Stiffness and Adhesion Receptor–Ligand Affinity
Source: Front Bioeng Biotechnol. 2020 Jun 3;8:459. doi: 10.3389/fbioe.2020.00459 (PMC7283468; doi:10.3389/fbioe.2020.00459)
Supplement: Supplementary file 1 [file Data_Sheet_1.PDF]

Supplementary Material

1 Computational Method

The following subsections discuss model implementation in more detail than in the main text. All parameter values can be found in Table 1.

1.1 Cell anatomy

To demarcate lamellipodium (Lp) and lamellum (Lm) along the cell-substrate interface, the cellular perimeter was first identified. Triangles in the perimeter acted as sources of actin. Actin diffused across all other triangles with a diffusion constant (D_{actin}) in an act representative of actin retrograde flow. Together with diffusion, the concentration of actin at each triangle i ($G_{\Delta i}$) (units of molecules/μm^2) was determined by generation at perimeter and degradation of actin; all triangles acted as sinks for actin. This effectively created a gradient of actin concentration that was highest at the edge and decayed along the bottom and top surfaces of the cell. The change in concentration of actin at each triangle is given by:

$$\frac{\delta [G]_{\Delta i}}{\delta t} = k_{gen,\Delta i} - k_{deg}[G]_{\Delta i} - D_{actin}\nabla^2 [G]_{\Delta i}, \tag{1}$$

where $k_{gen,\Delta i}$ is the rate at which actin is generated per surface area unit at the edge triangles of the cell, and k_{deg} is the rate at which actin is degraded in all triangles. The third term in the right-hand side of the equation corresponds to diffusion across the cell surface (i.e. across the sides of the triangular element i in the 2D mesh) according to Fick's second law. Diffusion is implemented as a forward Euler first order finite volume scheme in two dimensions that guarantees conservation of mass; this simple scheme suffices because the timestep is restricted by the fast mechanical relaxation time. By setting two threshold values the Lp and Lm areas were dynamically defined in simulations, $[G]_{Lp}$ and $[G]_{Lm}$: At every time step the concentration of actin at each triangle i was checked, if $[G]_{\Delta i} > [G]_{Lp}$ then the triangle was part of the Lp, and if $[G]_{Lp} > [G]_{\Delta i} > [G]_{Lm}$ then the triangle was part of the Lm. If $[G]_{\Delta i} < [G]_{Lp}$ the triangle was considered part of the cell body.

Additionally, front and rear halves of the cell were defined according to the position of the cell nodes at the cell-substrate interface. The center of mass (CoM) of the nodes was calculated; nodes with a higher x-position were part of the cell front, while the rest were part of the rear. Within front and rear halves only certain areas of the cell-substrate are considered active, such that the continuous Lp and

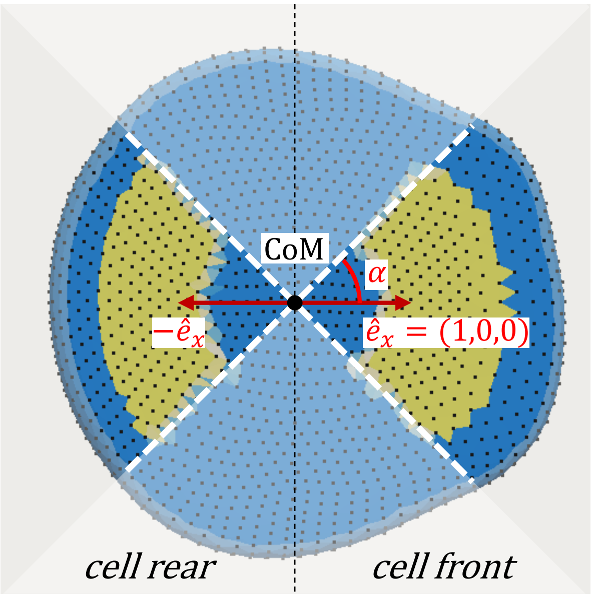

Fig. S1: Determination of cell polarization. View of nodes in cell-substrate interface and corresponding center of mass (CoM). Black dashed line separates front and rear cell halves. White dashed lines separate active (colored) and inactive (opaque) areas. Resulting active Lm shown in yellow and the rest of the interface in blue.

Lm are divided into a front and rear sections. These are asymmetric if the cell-substrate interface is asymmetric. Definition of the CoM and active areas of the cell was done according to the following steps:

1. Calculate CoM of nodes within 1 μm of the substrate
2. Vectors $-\hat{\boldsymbol{e}}_x = (-1, 0, 0)$ and $\hat{\boldsymbol{e}}_x = (1, 0, 0)$ are defined with origin at CoM, pointing at front and rear ends of the cell
3. Triangles (and corresponding nodes) within an angle α of these vectors are considered “active”

An illustration of this process of defining active areas can be found in Figure S1. The distinct areas and functional subcellular elements (i.e. focal adhesions (FAs) and stress fibers (SFs)) can be seen in Figure S2. Although in actual cells the Lp is only found in the protruding front of the cell, by using a threshold actin value, the entire perimeter is marked as Lp. This was done for tractability in the implementation. The protrusive activity, however, is reserved only to the active region (defined by angle α) in the front of the cell.

1.2 Discrete adhesions and substrate stiffness

FAs were modeled as discrete elements at the cell nodes, implemented through a two-spring system (in series), as proposed by Schwarz et al. [1]: A stiff spring

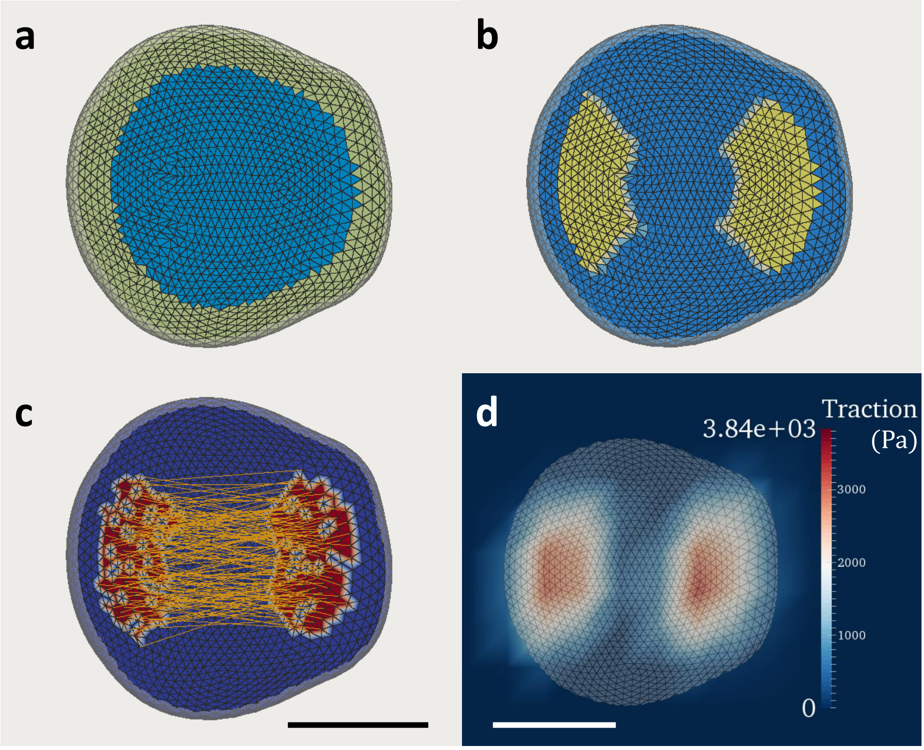

Fig. S2: Illustration of distinct cellular parts. Bottom view of cell-substrate interface showing: a) Lp area (green); b) distinct front and rear active Lm (yellow); c) FAs (red) in the active Lm, which can be connected by SFs (orange). There is an protrusion force at the front active Lp extending the cellular front. Probability of FA disassembly was 2× higher in rear Lm compared to front leading to retraction of the rear. d) Traction map of contracting cell before rupture and retraction. Scalebars 10 μm.

represented the FA (k_{FA}), while a softer spring represented the underlying ligand molecule (k_{ECM}).

The spring stiffness for the ligand (k_{ECM}) can be converted to a bulk stiffness felt locally by the cell value according to the following equation, taken from Mitrossilis et al. [2]:

$$E_{ECM} = \frac{k_{ECM}(1-\nu)^2}{D}, \tag{2}$$

where E_{ECM} is the Young's modulus of the substrate, ν is its Poisson's ratio, and D is the diameter of the contact area of the FA with the substrate. ν was set to 0.5, and D was set to 1 μm (which approximates the variable diameter observed throughout simulations).

1.3 Disassembly rate and expected lifetime

To arrive at a relation between FA disassembly rate ($r^0_{off,FA}$) and expected lifetime ($\langle\lambda^0_{FA}\rangle$) under no force, we note that FA disassembly is modeled as a Poisson process.

Our starting point is the survival function or complementary cumulative distribution function ($S(t)$) which gives the probability that an object of interest (e.g. patient, device, etc.) will survive beyond a specified time. The survival function for a continuous random variable T with cumulative distribution function $F(t)$ on the interval $[0,\infty)$ is defined according to:

$$S(t) = P(T > t) = \int_t^{\infty} f(u)\,\mathrm{d}u = 1 - F(t), \tag{3}$$

Because for FAs we are interested not in survival (i.e. probability of surviving beyond a time T), but rather death (i.e. probability of disassembly before a time T), the probability we are interested in is defined by $F(t)$ or $1 - S(t)$.

For a Poisson process, the cumulative distribution of a random variable T is given according to:

$$F(x;\lambda) = \begin{cases} 1 - e^{-\lambda x} & x \geq 0, \\ 0 & x < 0, \end{cases} \tag{4}$$

where λ is the rate parameter of the distribution, describing the average number of events in an interval ($\lambda > 0$). A property of the Poisson distribution is that λ is related to the expected value of the distribution $E[X]$ (for random variable X) through the following equation:

$$\lambda = \frac{1}{E[X]} \tag{5}$$

Substituting Eq. (5) into Eq. (4), we arrive at the following cumulative distribution function in terms of expected value:

$$F(x;\lambda) = 1 - e^{\frac{-x}{E[x]}} \equiv F(x)\ for\ x \geq 0. \tag{6}$$

Considering now $x \equiv t$ and solving for the expected value $E[t]$ yields,

$$E[t] = \frac{-t}{ln(1 - F(t))}, \tag{7}$$

where $F(t)$ is the probability of disassembly in particular time t. If we set this value $t = 1s$, then we can use the equivalency $F(t) \equiv r^0_{off,FA}$ (rate of disassembly of FA in 1s under no force). In which case the expected value ($E[t]$) corresponds to the expected lifetime of FAs under no force or $\langle\lambda^0_{FA}\rangle$:

$$E[t] = \langle\lambda^0_{FA}\rangle = \frac{-1}{ln(1 - r^0_{off,FA})}, \tag{8}$$

and we have thus arrived at Eq. 4 in the main text, describing the expected lifetimes of FAs for the different adhesion receptor-ligand affinity values ($r^0_{off,FA}$).

This can be corroborated by running a simulation in which 600 FAs (the approximate number of FAs in the simulated cell lamella) are allowed to form with

a rate $r_{on,FA} = 5e^{-3}s^{-1}$ and disassemble with the different $r^0_{off,FA}[s^{-1}]$ values used in the simulations for 24h (i.e. simulation time). The distribution of the lifetimes of the different FAs during this period are shown in Figure S3.

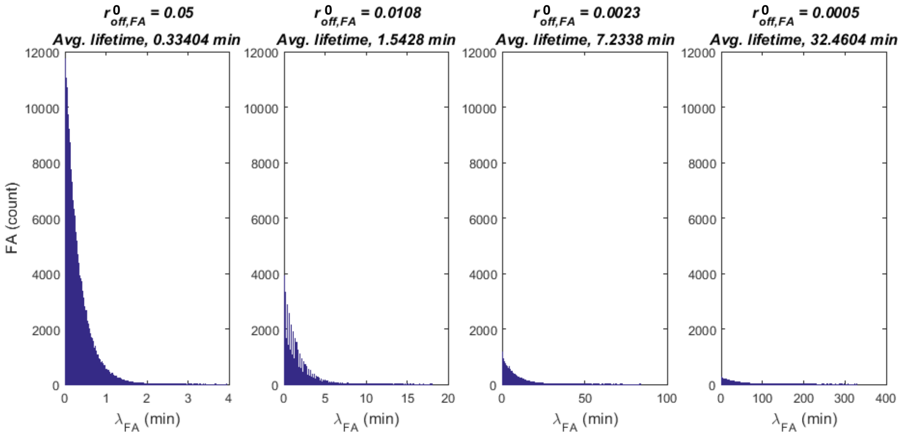

Fig. S3: Distribution of focal adhesion (FA) lifetime (λ_{FA}) for simulated FAs forming and disassembling over 24h. The different disassembly rates ($r^0_{off,FA}$) used correspond to those used in simulations of cell migration.

These distributions reveal that the average expected lifetime corresponds to that predicted by Eq. (8). Additionally, they show the relative number of FAs that are expected to live more and less than the expected average value.

1.4 Cortex elasticity

Assuming small deformations, we approximated in-plane elastic behavior of the cortex using linear springs. This led to a discrete spring network representation of a visco-elastic sheet. The force carried by the springs is given by:

$$\boldsymbol{F}^s_{ij} = k_{cortex}(d_{ij} - d^*_{ij})\hat{\boldsymbol{n}}_{ij}, \tag{9}$$

with

$$\hat{\boldsymbol{n}}_{ij} = \frac{\boldsymbol{x}_j - \boldsymbol{x}_i}{\|\boldsymbol{x}_j - \boldsymbol{x}_i\|} \tag{10}$$

Here, $d_{ij} = \|\boldsymbol{x}_j - \boldsymbol{x}_i\|$ is the current distance and d^*_{ij} the resting distance between nodes i and j with positions $\boldsymbol{x}_i$ and $\boldsymbol{x}_j$. Assigning a single stiffness value to all springs, however, does not simulate a uniform elastic cell membrane in equilibrium calculations. Therefore the linear spring stiffness k_{cortex} can be expressed as a function of an effective Young's modulus (E_c) and membrane thickness (t_c) in accordance with Van Gelder [3] – see Figure S4(a)

$$k_{cortex} = \frac{2\sqrt{3}}{3}E_c t_c, \tag{11}$$

by using this expression we have implicitly assumed a near uniform spring mesh.

Due to its non-zero thickness, the cortex also has bending rigidity. The energy required to bend two connected triangles ($\alpha\beta$) is given by:

$$E^b_{\alpha\beta} = k_{bend}\left(1 - \cos\left(\theta - \theta^*\right)\right), \tag{12}$$

where θ^* and θ represent the spontaneous and instantaneous angles between a pair of adjacent triangles.

As with k_{cortex}, bending rigidity k_{bend} can be estimated based on cortex properties to match to macroscopic (continuum) models. Based on the model of Helfrich [4]:

$$k_{bend} = \frac{E_c t_c^3}{12\left(1 - \nu_c^2\right)}, \tag{13}$$

with ν_{cortex} as the Poisson's ratio of the cortex. For a pair of connected triangles $\alpha\beta$, the bending moment is:

$$\boldsymbol{M}^b_{\alpha\beta} = -k_{bend}(\theta - \theta^*)\frac{\boldsymbol{x}^{c2}_{\alpha\beta} - \boldsymbol{x}^{c1}_{\alpha\beta}}{\left\|\boldsymbol{x}^{c2}_{\alpha\beta} - \boldsymbol{x}^{c1}_{\alpha\beta}\right\|}, \tag{14}$$

for small angle deviations when $\sin\left(\theta - \theta^*\right) \approx \theta - \theta^*$, and with $\boldsymbol{x}^{c1}_{\alpha\beta}$ and $\boldsymbol{x}^{c2}_{\alpha\beta}$ the positions of the connected triangles' common nodes, sorted counter-clockwise with respect to the triangle normal vectors $\hat{\boldsymbol{n}}_\alpha$ and $\hat{\boldsymbol{n}}_\beta$ – see Figure S4(b). This couple is translated to mechanically equivalent forces on the four nodes of the triangle pair. For each triangle, the sum of all three forces must be zero, and the generated moment w.r.t. the common axis must be $\boldsymbol{M}^b_{\alpha\beta}$. These conditions lead to following unique total nodal forces

$$\boldsymbol{F}^{h_\alpha}_{\alpha\beta} = -\boldsymbol{M}^b_{\alpha\beta} \times \boldsymbol{h}_\alpha, \tag{15}$$

$$\boldsymbol{F}^{h_\beta}_{\alpha\beta} = \boldsymbol{M}^b_{\alpha\beta} \times \boldsymbol{h}_\beta, \tag{16}$$

$$\boldsymbol{F}^{c1}_{\alpha\beta} = \frac{y^{c2}_\beta}{y^{c1}_\beta - y^{c2}_\beta}\left(\boldsymbol{M}^b_{\alpha\beta} \times \boldsymbol{h}_\beta\right) - \frac{y^{c2}_\alpha}{y^{c1}_\alpha - y^{c2}_\alpha}\left(\boldsymbol{M}^b_{\alpha\beta} \times \boldsymbol{h}_\alpha\right), \tag{17}$$

$$\boldsymbol{F}^{c2}_{\alpha\beta} = \frac{y^{c1}_\beta}{y^{c2}_\beta - y^{c1}_\beta}\left(\boldsymbol{M}^b_{\alpha\beta} \times \boldsymbol{h}_\beta\right) - \frac{y^{c1}_\alpha}{y^{c2}_\alpha - y^{c1}_\alpha}\left(\boldsymbol{M}^b_{\alpha\beta} \times \boldsymbol{h}_\alpha\right). \tag{18}$$

h_α and h_β indicate the indices of the (non-common) "lever" node of triangles α and β. $\boldsymbol{h}_\alpha$ and $\boldsymbol{h}_\beta$ are the orthogonal height vectors from the common axis to lever nodes h_α and h_β. Finally,

$$y^{cj}_k = (\boldsymbol{x}^{cj}_{\alpha\beta} - \boldsymbol{x}^{h_k}_{\alpha\beta}) \cdot (\boldsymbol{x}^{c2}_{\alpha\beta} - \boldsymbol{x}^{c1}_{\alpha\beta}), \tag{19}$$

for $k \in [\alpha, \beta]$ and $j \in [1, 2]$.

Additionally, local and global area constraints are implemented, ensuring that both individual triangle areas and total cell area do not change drastically. These constraints are implemented by computing a tension for each triangle ($T_\triangle$), described according to:

$$T_\triangle = k_{A,local}(A_\triangle - A^*_\triangle)/A^*_\triangle + T_g/2, \tag{20}$$

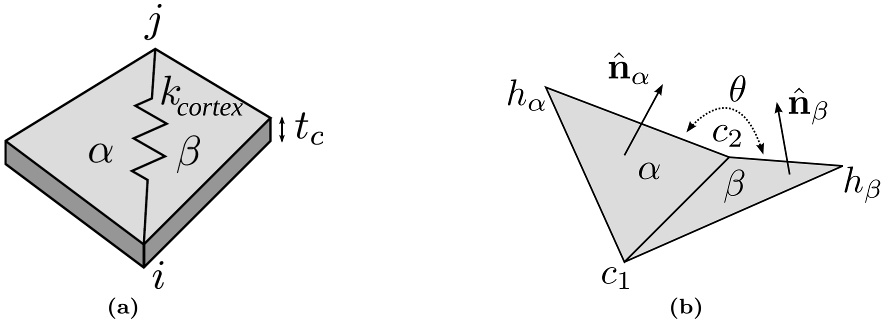

Fig. S4: **a)** Illustration of elementary spring element between nodes i and j. The spring constant k_{cortex} is based on a thin shell element containing adjacent triangles α and β, and with thickness t_c – Eq. (11). **b)** Illustration of two connected triangles α and β with normal unit vectors $\hat{\boldsymbol{n}}_\alpha$ and $\hat{\boldsymbol{n}}_\beta$ between whom a bending moment is computed based on the instantaneous angle θ. Furthermore, we have indicated (sorted) common nodes c_1 and c_2 and lever nodes h_α and h_β.

where $A_\triangle$ is the area of the triangle, $A^*_\triangle$ the resting area of the triangle, $k_{A,local}$ the constraint constant, and T_g a global cell tension describing the global constraint. T_g is described by the following:

$$T_g = k_{A,global}(A_{tot} - A^*_{tot}), \tag{21}$$

where A_{tot} is the total cell area, A^*_{tot} the total resting area, and $k_{A,global}$ the constraint constant. The resulting tension ($T_\triangle$) per triangle is converted to a force acting on the triangle nodes in the direction towards the opposite triangle side: For each node i on the triangle with normal vector $n_\triangle$, the force contribution is,

$$\boldsymbol{F}_{A,i} = T_\triangle(\boldsymbol{x}_k - \boldsymbol{x}_j) \times \hat{\boldsymbol{n}}_\triangle. \tag{22}$$

Finally, we add a volume constraint: As the equilibrium volume of a cell is assumed to be constant at short time-scales, an effective bulk modulus k_{vol} is introduced. The cytoplasmic pressure due to volume control P^v can thus be estimated as

$$P^v = -k_{vol}\frac{V - V^*}{V^*}. \tag{23}$$

V^* and V represent the spontaneous and instantaneous volume of the cell. The resulting nodal force due to the total internal pressure P^v is given by

$$\boldsymbol{F}_{vol} = \hat{\boldsymbol{n}}_i \mathcal{A}_i P^v, \tag{24}$$

with $\mathcal{A}_i$ and $\hat{\boldsymbol{n}}_i$ the Voronoi area [5] and normal associated with a given node.

1.5 Contact mechanics

In our numerical model, we represent deformable cells as triangulated meshes, where the local curvature is taken into account for each triangle by means of an

encompassing sphere. The contact between two rounded triangles can be modeled by using the Maugis-Dugdale (MD) theory for overlapping spheres [5].

MD expands upon Hertz' pure repulsive contact model by taking into account the adhesive pressure associated with intimate contact between adherent surfaces – see Figure S5. The MD contact pressure between two curved asperities A and B is given by the sum of Hertz and adhesive pressures:

$$p(r) = p_a(r) + p_H(r). \tag{25}$$

The repulsive Hertz pressure acting on the contact area with radius a, for a given distance r from the center of the contact circle, is given by

$$p_H(r) = \frac{2\hat{E}_{AB}}{\pi \hat{R}_{AB}} \sqrt{a^2 - r^2}, \tag{26}$$

with effective Young's modulus $\hat{E}_{AB}$ and contact radius $\hat{R}_{AB}$

$$\hat{E}_{AB} = \left(\frac{1-\nu_A^2}{E_A} + \frac{1-\nu_B^2}{E_B} \right)^{-1},$$

$$\hat{R}_{AB} = \left(\kappa_A + \kappa_B\right)^{-1},$$

where E_A, ν_A and κ_A refer to the Young's modulus, Poisson's ratio and local curvature of a given asperity A. Adhesive stress is given by

$$p_a(r) = \begin{cases} -\frac{\sigma_0}{\pi} \arccos\left(\frac{2a^2-c^2-r^2}{c^2-r^2}\right) & 0 < r < a, \\ -\sigma_0, & a < r < c, \end{cases} \tag{27}$$

Here, σ_0 represents the maximal adhesive traction, which is related to the adhesion energy W as [7]:

$$W = h_0\, \sigma_0, \tag{28}$$

where h_0 represents the maximum separation between the asperities beyond which the adhesive traction drops to zero.

Numerical integration of the contact pressures allows us to determine the net contact force and moment acting on a pair of triangles $(\alpha\beta)$. Assuming that the nodal contact forces $F_i^{\alpha\beta}$ must be colinear with the contact unit normal $\hat{\boldsymbol{n}}_{\alpha\beta}$, the system of linear equations per contact pair $(\alpha\beta)$:

$$\sum_{i\in\alpha} \boldsymbol{F}_i^{\alpha\beta} = -\sum_{q\in\alpha\cap\beta} A_q\, p(\|\boldsymbol{r}_q\|)\hat{\boldsymbol{n}}_{\alpha\beta}, \tag{29}$$

$$\sum_{i\in\alpha} \left[\boldsymbol{x}_i - \boldsymbol{x}_C^{\alpha\beta} + \left[(\boldsymbol{x}_C^{\alpha\beta} - \boldsymbol{x}_i)\cdot\hat{\boldsymbol{n}}_{\alpha\beta}\right]\hat{\boldsymbol{n}}_{\alpha\beta}\right] \times \boldsymbol{F}_i^{\alpha\beta} = -\sum_{q\in\alpha\cap\beta} A_q p(\|\boldsymbol{r}_q\|)\boldsymbol{r}_q \times \hat{\boldsymbol{n}}_{\alpha\beta}, \tag{30}$$

results in a unique solution for every $\boldsymbol{F}_i^{\alpha\beta}$. A_q is the weighted area associated with quadrature points q, covering the intersection polygon $\alpha\cap\beta$. $\boldsymbol{r}_q$ is the vector from the sphere-sphere[1] contact point $\boldsymbol{x}_C^{\alpha\beta}$ to the quadrature point. The solution for this system is presented in Odenthal et al. [5].

[1] Each triangle with curvature κ can be associated with a unique sphere with radius $1/\kappa$, see [5].

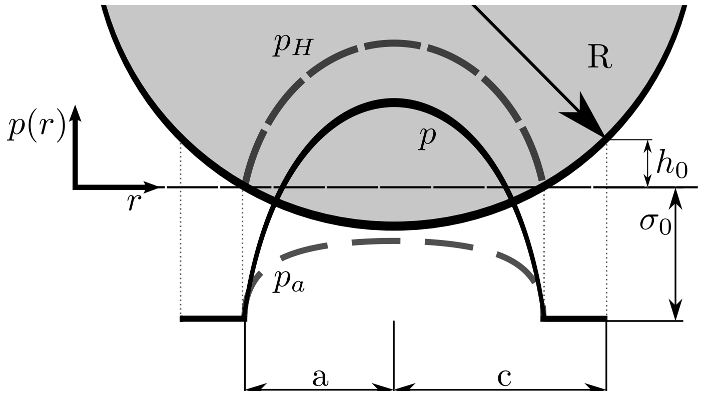

Fig. S5: Illustration of contact between an asperity with radius R and a flat half-space. The total contact pressure p is the sum of the repulsive Hertz pressure p_H, acting within contact radius a and an adhesive Dugdale traction p_a, acting within contact radius c. For $a \leq r \leq c$, the adhesive traction is at its maximal value σ_0.

1.6 Dissipative forces

Our method is based on solving overdamped equations of motion. Hence, dissipative forces are required to balance these equations of motion. The general methodology tries to introduce these dissipative forces in a consistent manner in terms of viscosities of modeled materials. A general drag force $\boldsymbol{F}_i^l$ is included to account for the liquid drag between the cells and their medium:

$$\boldsymbol{F}_i^l = -\lambda_l \mathcal{A}_i \boldsymbol{v}_i, \tag{31}$$

with $\mathcal{A}_i$ the Voronoi area of node i. For spherical cells with radius R

$$\lambda_l = \frac{3\eta_l}{2R} \tag{32}$$

can be used to estimate λ_l, introducing fluid viscosity η_l. When dealing with arbitrary shapes this approximation is no longer correct and we would in principle require the microscopic resolution of the fluid flow field in and around the cell surface. However, as F_i^d is typically very small compared to other dissipative forces at the seconds/minutes timescale, this approximation is sufficient. Likewise, we can increase the fluid viscosity above realistic values to dampen numerical oscillations without any influence on simulation results. A much larger contribution to energy dissipation arises from viscosity of the cortex itself. The viscous damping force between two connected nodes i and j is computed as

$$\boldsymbol{F}_{ij}^d = \Lambda^d (\boldsymbol{v}_j - \boldsymbol{v}_i), \tag{33}$$

with friction elements ($\boldsymbol{I}$ being identity)

$$c = \frac{t_c\, \eta_c}{\sqrt{3}}\, \boldsymbol{I}.$$

where the $1/\sqrt{3}$ factor accounts for the triangular connectivity of the shell. Finally, a viscous contact force is included to account for drag between contacting triangles. The contact drag force acting on node i of triangle α of the contacting pair $(\alpha\beta)$

$$\boldsymbol{F}^{c}_{\alpha\beta,i} = \Lambda^{c}_{\alpha\beta} \cdot \sum_{\forall k \in \beta} w_{\alpha\beta,ik} \, (\boldsymbol{v}_k - \boldsymbol{v}_i), \tag{34}$$

again, determined by a friction tensor $\Lambda^{c}_{\alpha\beta}$ and weights $w_{\alpha\beta,ik}$ per node k of the β triangle. $w_{\alpha\beta,ik}$ are assumed to scale with the relative contribution of the nodal contact forces to the overall contact force, thus

$$w_{\alpha\beta,ik} = \frac{(\boldsymbol{F}_{\alpha\beta,i} + \boldsymbol{F}_{\alpha\beta,k}) \cdot \hat{\boldsymbol{n}}_{\alpha\beta}}{6 \sum\limits_{\forall k \in \beta} \boldsymbol{F}^{MD}_{\alpha\beta,k} \cdot \hat{\boldsymbol{n}}_{\alpha\beta}}, \tag{35}$$

$\Lambda^{c}_{\alpha\beta}$ for a given contact area $A^{c}_{\alpha\beta}$ between triangles α and β is estimated as:

$$\Lambda^{c}_{\alpha\beta} = A^{c}_{\alpha\beta} \left[\lambda_n \hat{\boldsymbol{n}}_{\alpha\beta} \cdot \hat{\boldsymbol{n}}^{T}_{\alpha\beta} + \lambda_t \left(\boldsymbol{I} - \hat{\boldsymbol{n}}_{\alpha\beta} \cdot \hat{\boldsymbol{n}}^{T}_{\alpha\beta} \right) \right], \tag{36}$$

with normal and tangential friction coefficients[2] λ_n and λ_t.

In the main text (Equation 11), the weights of the nodes to each triangle when taking into account area are omitted for clarity, and the product of the friction tensor $\Lambda^{c}_{\alpha\beta}$ and weights $w_{\alpha\beta,ik}$ are presented as a single element Γ: For cell-substrate contact this is Γ_{subs} (calculated using friction coeffiecients $\lambda_{t,cs}$ and $\lambda_{n,cs}$).

1.7 Equation of motion

Neglecting inertial contributions for the overdamped cellular system, the complete force balance for node i can be expressed based on the different contributions described above

$$\sum_{\text{con}.j} \boldsymbol{F}^{s}_{ij} + \sum_{(\alpha\beta):i\in\alpha} \boldsymbol{F}^{\alpha\beta}_{i} + \sum_{(\alpha\beta):i\in\alpha} \boldsymbol{F}^{b}_{\alpha\beta,i} + \boldsymbol{F}^{p}_{i}$$

$$= \tag{37}$$

$$\sum_{\text{con}.j} \Lambda^{d} \cdot w_{ij} \, (\boldsymbol{v}_i - \boldsymbol{v}_j) + \sum_{(\alpha\beta):i\in\alpha} \left[\Lambda^{c}_{\alpha\beta,i} \cdot \sum_{\forall k \in \beta} w_{\alpha\beta,ik} \, (\boldsymbol{v}_i - \boldsymbol{v}_k) \right] + \lambda^{l} \mathcal{A}_i \boldsymbol{v}_i,$$

For a system of N nodes, Eq. (37) can be summarized as:

$$\underline{\boldsymbol{F}} = \underline{\Lambda} \cdot \underline{\boldsymbol{v}}, \tag{38}$$

[2] Note that the units of friction coefficient λ_n and λ_t are Pa·s/m, as they relate a velocity difference between two contacting surface to a dissipative contact stress.

which consist of a $(3N \times 1)$, $(3N \times 3N)$ and $(3N \times 1)$ matrix for three-dimensional systems. $\underline{\Lambda}$ is a symmetric and positive definite matrix

$$\underline{\Lambda} = \sum_{i,j \in N} \begin{pmatrix} 0 & \cdots & & & \\ \cdots & \Lambda_{ij} & \cdots & -\Lambda_{ij} & \cdots \\ \vdots & \vdots & \ddots & \vdots & \\ \cdots & -\Lambda_{ij} & \cdots & \Lambda_{ij} & \cdots \\ & & & \cdots & 0 \end{pmatrix} + \begin{pmatrix} \lambda_l & 0 & \cdots & & \\ 0 & \lambda_l & 0 & \cdots & \\ \vdots & & \ddots & & \vdots \\ & & 0 & \lambda_l & 0 \\ 0 & & \cdots & 0 & \lambda_l \end{pmatrix}, \tag{39}$$

where Λ_{ij} are (3×3) matrices created by $w_{ij} \Lambda^d_{ij} + w_{\alpha\beta,ij} \Lambda^c_{\alpha\beta_i}$. Since $\underline{\Lambda}$ is extremely sparse and always positive definite [5,6], the conjugate gradient method can be used to efficiently solve the system for nodal velocities $\underline{\boldsymbol{v}}(t)$ at each time increment. The positions of the nodes $\underline{\boldsymbol{x}}$ are subsequently updated using a forward Euler scheme:

$$\underline{\boldsymbol{x}}(t + \Delta t) = \underline{\boldsymbol{x}}(t) + \Delta t\, \underline{\boldsymbol{v}}(t). \tag{40}$$

1.8 Numerical solution and implementation

The computational model was implemented in the C++ particle-based simulation framework *Mpacts* (www.mpacts.com). The deformable cell model was first introduced in [5], and later expanded upon for shell mechanics in [8]. For solving over-damped systems, we use a semi-implicit method – see Eq. (38), where a friction matrix is assembled that contains contact friction (or stiffness) elements. Each timestep, this linear system is iteratively solved using the Conjugate Gradient implementation of the C++ linear algebra library Eigen [9] which is optimized for vectorization and performance. Furthermore, a multi-grid contact detection scheme [10] was used to efficiently resolve pairs of contacting triangles; the contact pressures were numerically integrated. Numerical integration was performed using a 7-point symmetric Gaussian quadrature rule as derived in [11]. Highly regular triangulated surface meshes of spherical cells were obtained by the progressive subdivision of an icosahedron – see e.g. [5]. We used 5-level subdivisions (resulting in 2562 vertices and 5120 triangles). 5-level subdivisions of the icosahedron are required to accurately model the high curvatures occurring when the cell is fully spread out. Using a smaller subdivision would result in a smaller number of FAs being formed and less force being exerted by the cell; also with this subdivision the area of the cell per node is approximately 0.785 μm^2, which is comparable to the area of a FA [12].

Each simulation was run using an Intel Xeon Processor E5-2680 v3 on a node with 2.7 GB of memory; simulations were run in parallel utilizing the multiple nodes (24) per core. Each simulation took approximately 24 h to run.

2 Additional Figures

Additional figure showing a graphical representation of the order of commands in the simulation loop (i.e. flowchart) can be found in Figure S6.

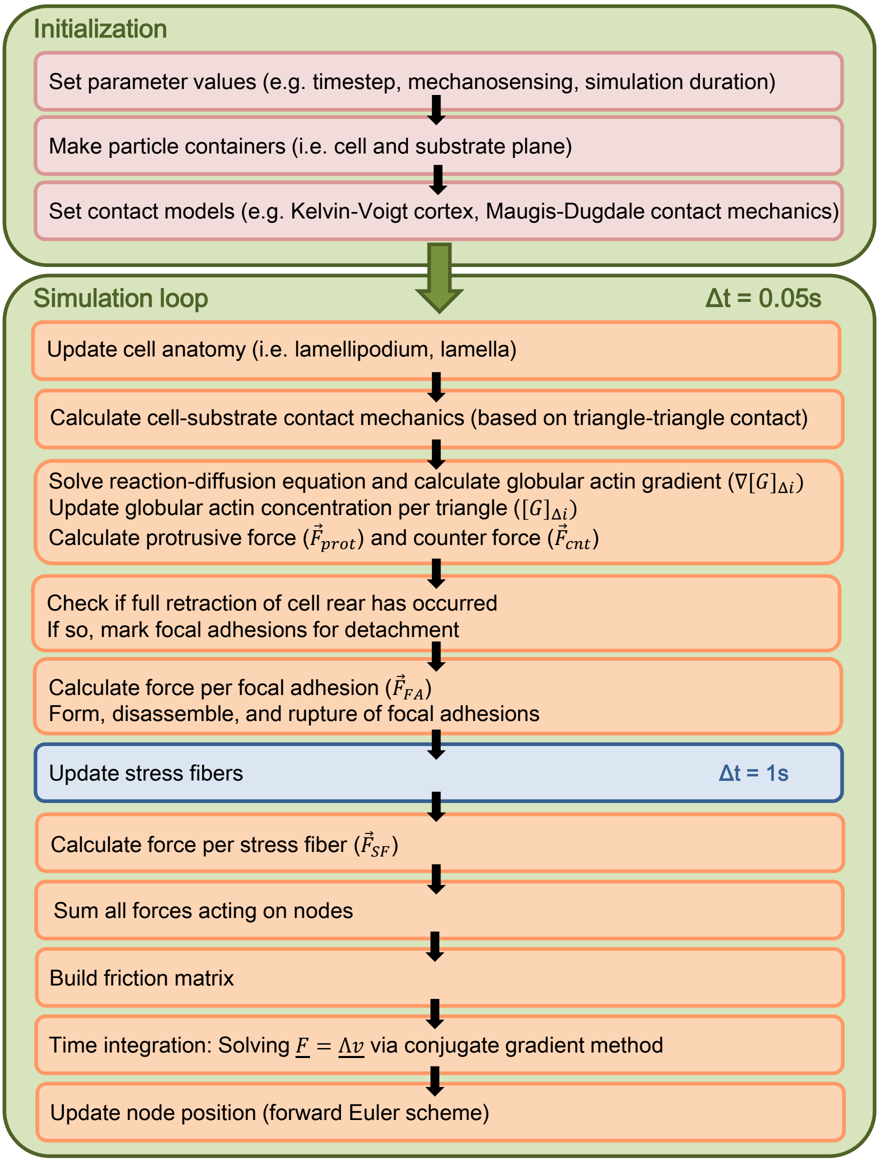

Fig. S6: Flowchart showing main commands and order of execution in the simulation loop. Initializaiton is run once, while commands in the simulation loop are run in every timestep with the exception of updating of stress fibers (run every 1s).

Additional figures showing values for the different output metrics calculated for every simulation and averaged over the replicates per condition (n=5): Displacement of the center of mass, actual lifetime of FAs, factor by which SFs strengthen ($n_{str,f}$), number of FAs, and cumulative number of ruptured FAs due to force.

These values correspond to those shown in the heatmaps in Figures 5-8; here results are presented in scatter plots to better show the relative variation between different replicates, displayed as error bars corresponding to the standard error of the mean (SEM). Values are shown for the 4 setups studies (defined by whether FA maturation (FA_{mat}) and SF strengthening (SF_{str}) were set to ON or OFF), and all conditions defined according to the values of substrate stiffness (k_{ECM}) and adhesion receptor-ligand affinity (λ^0_{FA}).

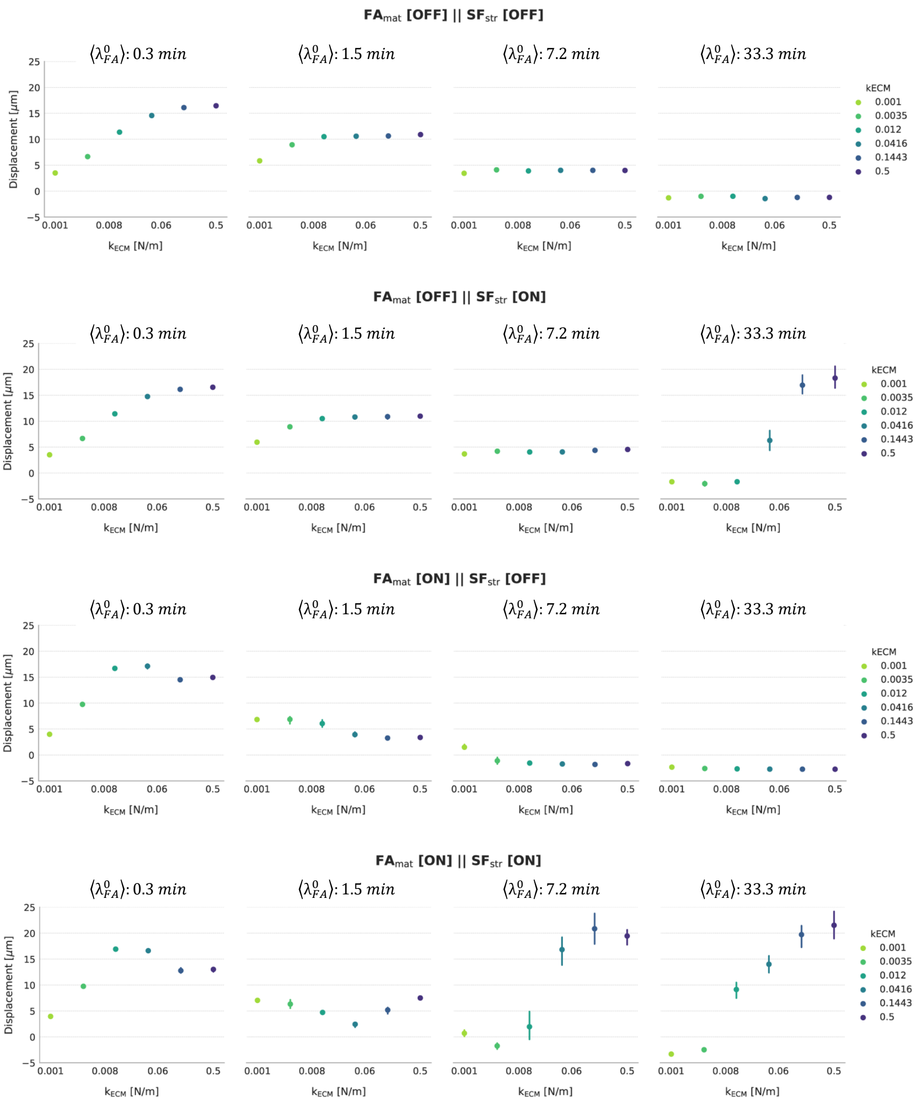

Fig. S7: Displacement of center of mass of nodes at cell substrate interface at the end of the each simulation for all setups and conditions. Averaged over replicates (n=5). Error bars correspond to standard error of the mean (SEM).

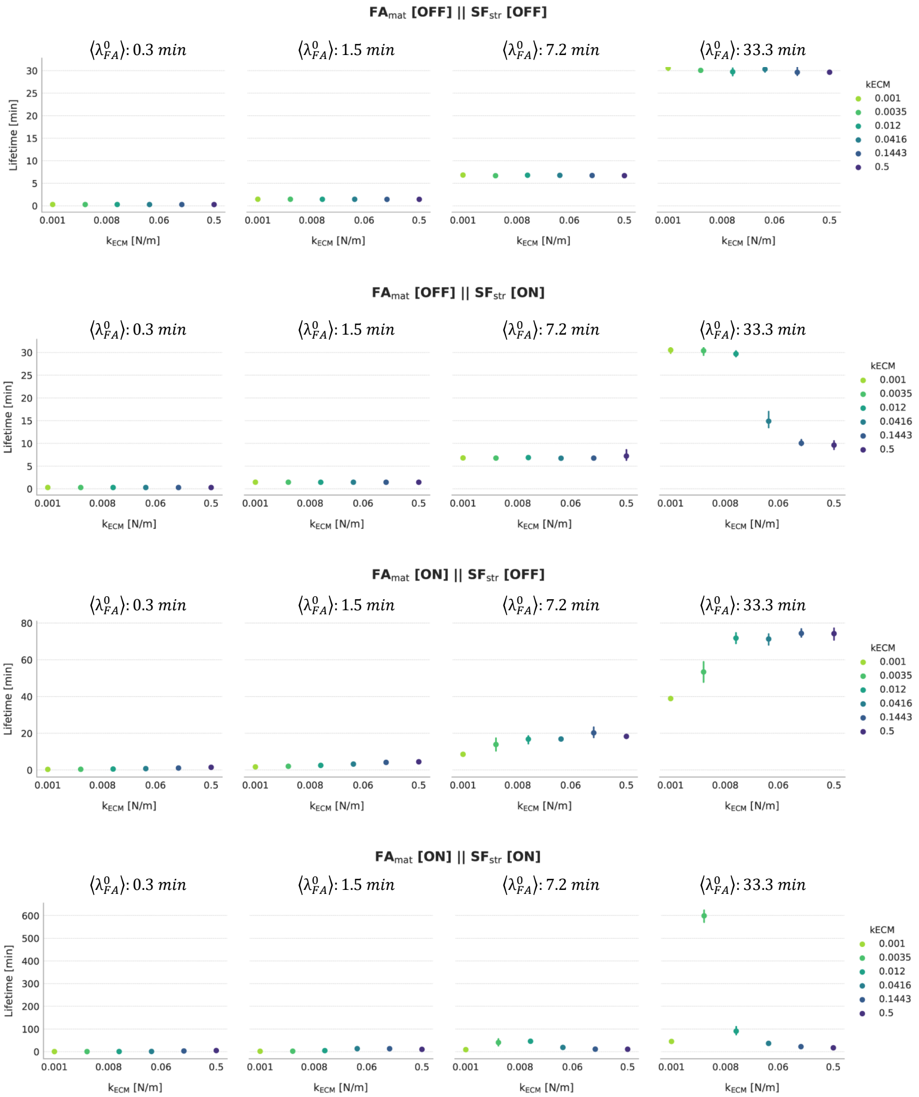

Fig. S8: Focal adhesion lifetime throughout simulations for all setups and conditions. Averaged over replicates (n=5). Error bars correspond to standard error of the mean (SEM).

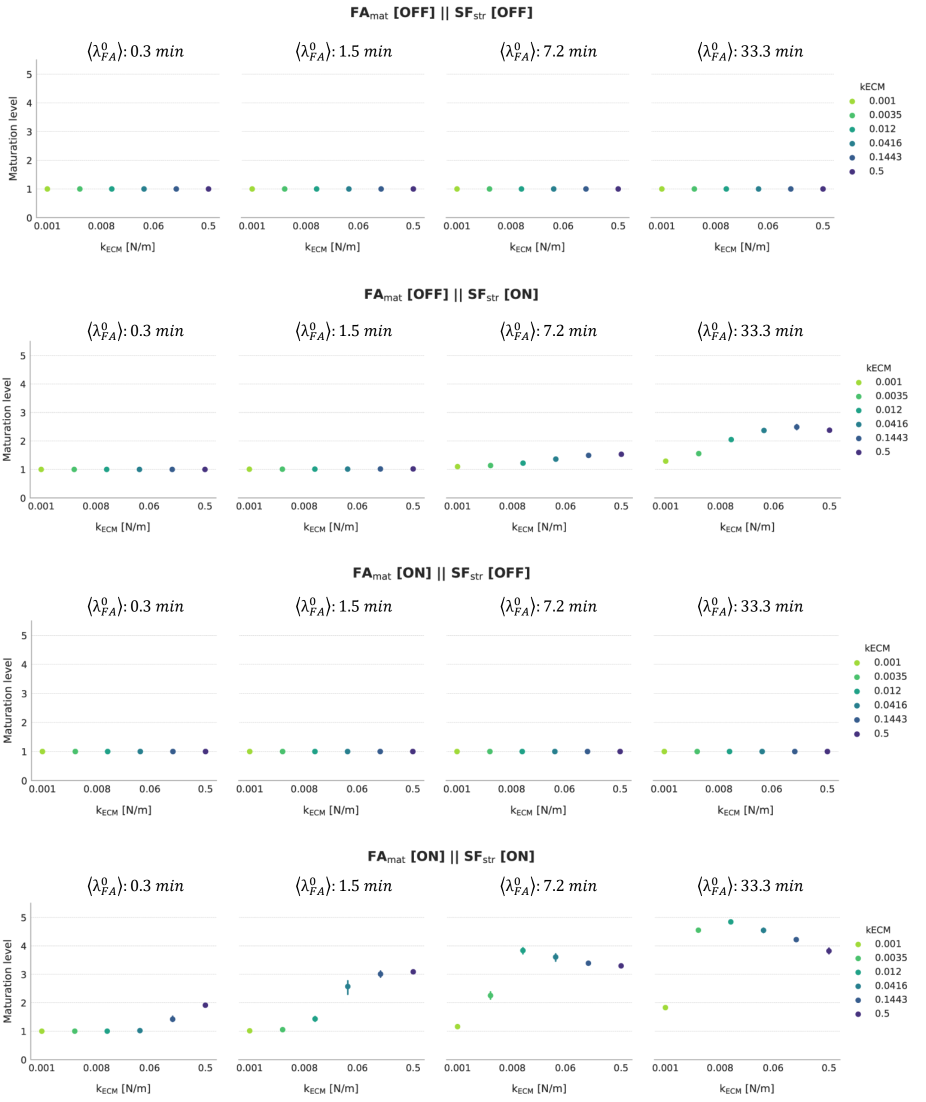

Fig. S9: Stress fiber strengthening factor ($n_{str,f}$) due to stalling throughout simulations for all setups and conditions. Averaged over replicates (n=5). Error bars correspond to standard error of the mean (SEM).

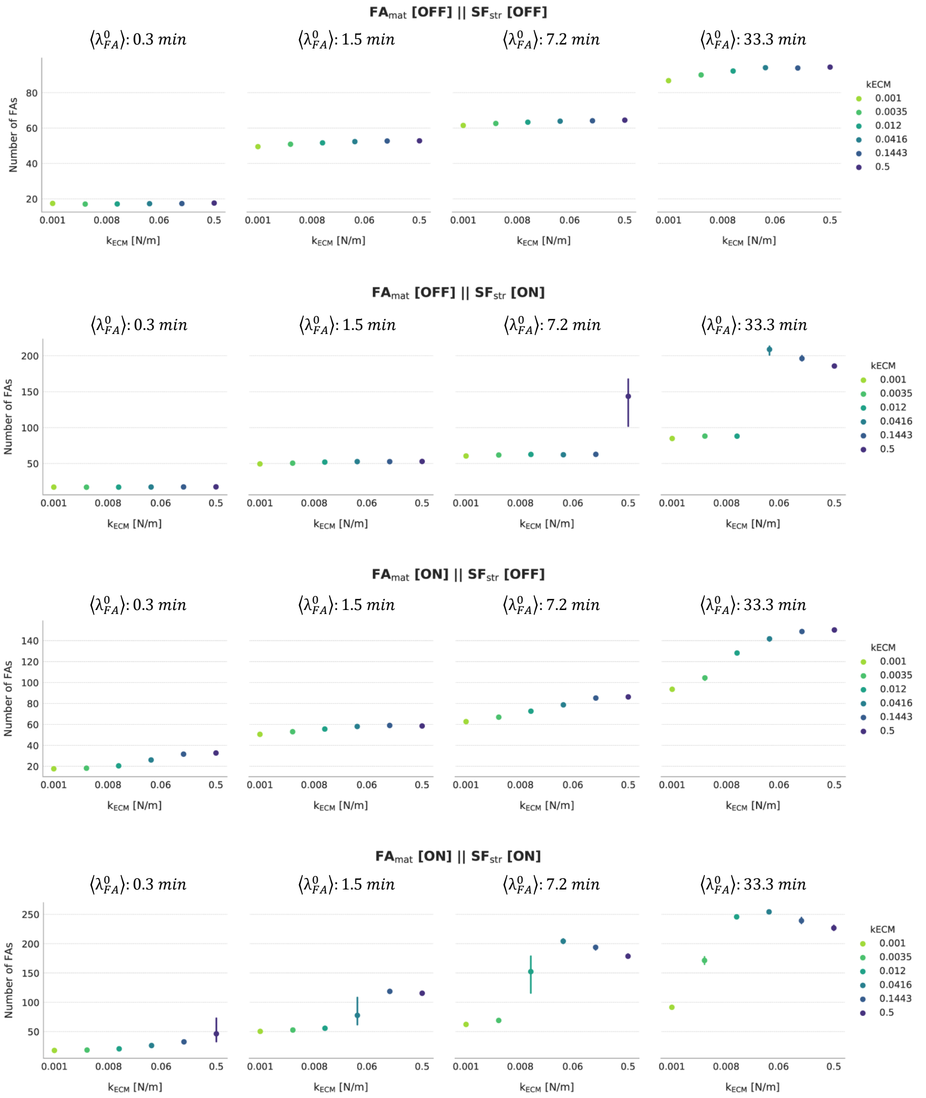

Fig. S10: Number of focal adhesions (FA) adhering the cell to the substrate throughout simulations for all setups and conditions. Averaged over replicates (n=5). Error bars correspond to standard error of the mean (SEM).

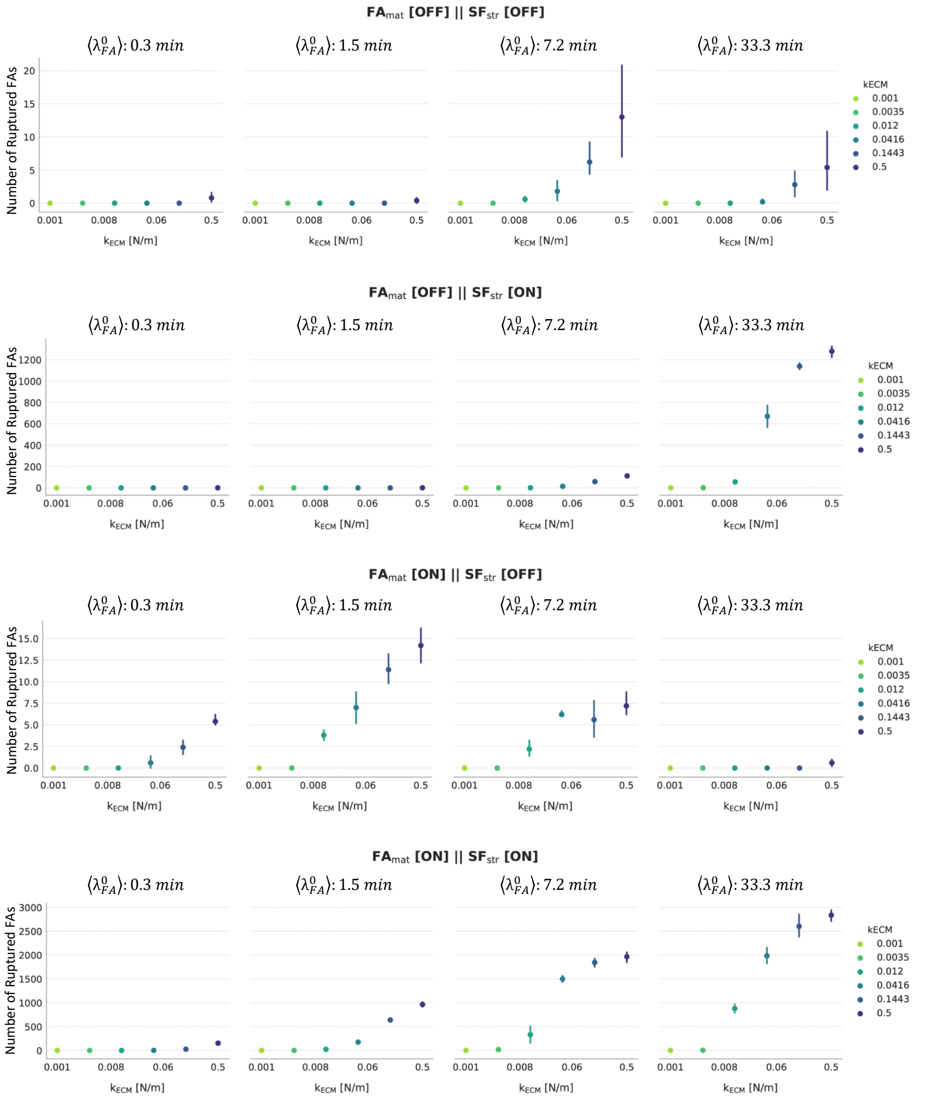

Fig. S11: Cumulative number of focal adhesions (FA) that ruptured due to excessive force throughout simulations for all setups and conditions. Averaged over replicates (n=5). Error bars correspond to standard error of the mean (SEM).

Additional figures that show quantification of differences in metrics describing cell migration in the different conditions (defined according to substrate stiffness (k_{ECM}) and expected lifetime of FAs under no force ($\langle\lambda\rangle^0_{FA}$)) for simulation setups with different mechanosensing mechanisms implemented.

Figure S12 shows the effect of SF strengthening (SF_{str}) by comparing both setups in which FA maturation (FA_{mat}) is OFF: $FA_{mat}[OFF]||SF_{str}[OFF]$ and $FA_{mat}[OFF]||SF_{str}[ON]$.

Figure S13 shows the effect of FA maturation (FA_{mat}), by comparing both setups in which FA maturation (FA_{mat}) is ON: $FA_{mat}[ON]||SF_{str}[OFF]$ and $FA_{mat}[ON]||SF_{str}[ON]$.

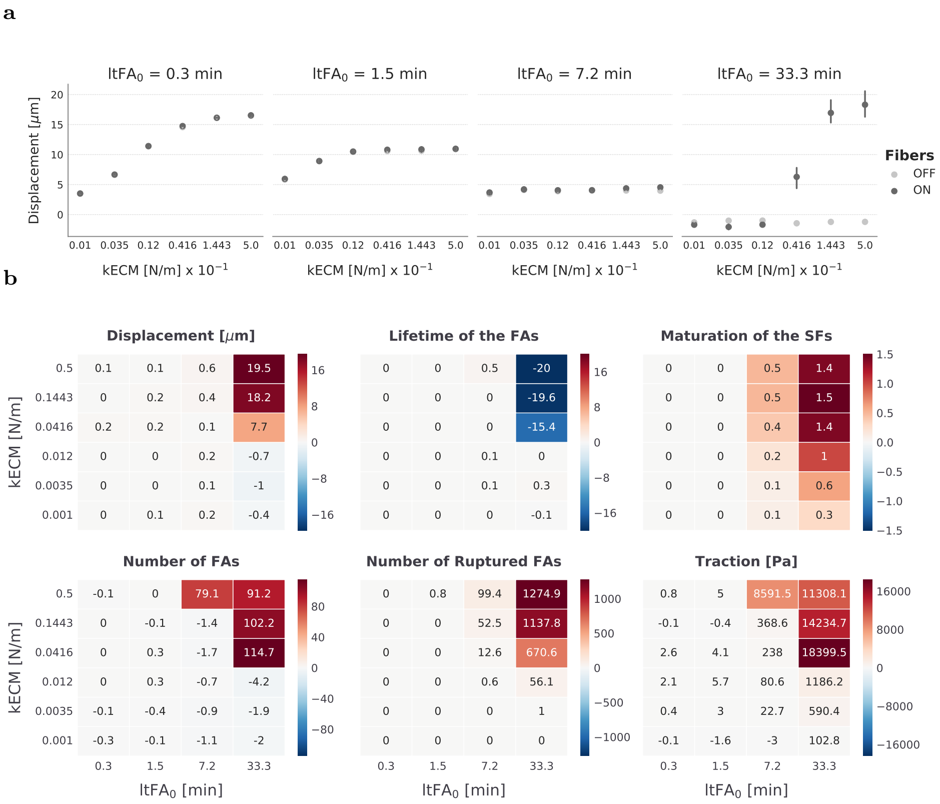

Fig. S12: Representation of the effect introduced by the strengthening of SFs. **a)** Final displacement values of the conditions with no maturation of the adhesions: $FA_{mat}[OFF]||SF_{str}[OFF]$ (light gray) and $FA_{mat}[OFF]||SF_{str}[ON]$ (dark gray), highlighting how strengthening only has an effect on displacement for $\langle\lambda\rangle^0_{FA} = 33.3$ min and $kECM \geq 0.416$ N/m. **b)** Differences between the two conditions for all the other metrics in study, further confirming that significant differences are only seen for high lifetimes and high stiffness values. Output metric values for the $SF_{str}[OFF]$ setup were subtracted from the $SF_{str}[ON]$ setup to obtain new values.

a

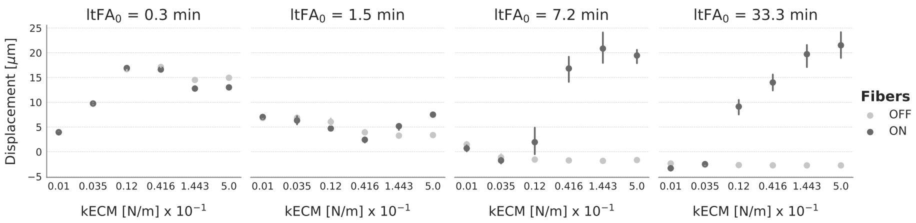

b

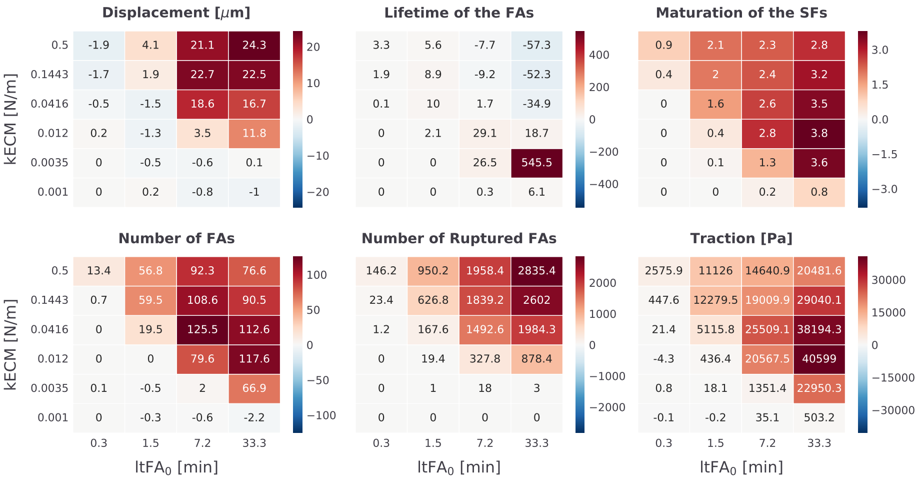

Fig. S13: Representation of the effect introduced by the maturation of the FAs. **a)** Final displacement values of the conditions with maturation of the adhesions: $FA_{mat}[ON]||SF_{str}[OFF]$ (light gray) and $FA_{mat}[ON]||SF_{str}[ON]$ (dark gray). **b)** Differences between the two conditions for all the other metrics in study. Output metric values for the $SF_{str}[OFF]$ setup were subtracted from the $SF_{str}[ON]$ setup to obtain new values.

3 Video Captions

Video S1. Animation of a migrating cell displaying progressive retraction (bottom view), showing cell shape and traction magnitude exerted on the substrate plane. It corresponds to cell that migrated the most during simulation time (24 h): condition $k_{ECM} = 0.0416$ N/m and $\langle\lambda\rangle^0_{FA} = 0.3$ min, setup $FA_{mat}[ON]||SF_{str}[ON]$. Link to animation: `https://figshare.com/articles/Video_S1/11604555`

Video S2. Animation of a migrating cell displaying progressive retraction (bottom view), showing how front and rear lamella (Lm)(yellow) change shape within the rest of the cell surface (blue) throughout a simulation. It corresponds to cell that migrated the most during simulation time (24 h): condition $k_{ECM} = 0.0416$ N/m and $\langle\lambda\rangle^0_{FA} = 0.3$ min, setup $FA_{mat}[ON]||SF_{str}[ON]$. Link to animation: `https://figshare.com/articles/Video_S2/11604591`

Video S3. Animation of a migrating cell displaying progressive retraction (bottom view), showing the position presence of focal adhesions (FAs)(red) at the cell-substrate interface. It corresponds to cell that migrated the most during simulation time (24 h): condition $k_{ECM} = 0.0416$ N/m and $\langle\lambda\rangle^0_{FA} = 0.3$ min, setup $FA_{mat}[ON]||SF_{str}[ON]$. Link to animation:
`https://figshare.com/articles/Video_S3/11604600`

Video S4. Animation of a migrating cell displaying progressive retraction (bottom view), showing the time elapsed since a stress fiber (SF) has detached from the substrate at one of its ends($\lambda_{detach,FA}$) before deletion. Most SFs are deleted approximately 7 min. after detachment, and occasionally after up to 14.9 min. It corresponds to cell that migrated the most during simulation time (24 h): condition $k_{ECM} = 0.0416$ N/m and $\langle\lambda\rangle^0_{FA} = 0.3$ min, setup $FA_{mat}[ON]||SF_{str}[ON]$. Link to animation: `https://figshare.com/articles/Video_S4/12094083`

Video S5. Animation of a migrating cell displaying progressive retraction (bottom view), showing the strengthening factor ($n_{str,f}$) per stress fiber (SF) throughout a simulation. Most fibers do not mature, while a few strengthen by a factor of 2 or 3. It corresponds to cell that migrated the most during simulation time (24 h): condition $k_{ECM} = 0.0416$ N/m and $\langle\lambda\rangle^0_{FA} = 0.3$ min, setup $FA_{mat}[ON]||SF_{str}[ON]$. Link to animation: `https://figshare.com/articles/Video_S5/12094098`

Video S6. Animation of a migrating cell displaying progressive retraction (bottom view), showing the time remaining for each node in the cell cortex in a refractory state (t_{ref}) before it can form a focal adhesion (FA) with the substrate below. Because rupture due to excessive force is rare, most nodes in a refractory state are just entering the lamella. It corresponds to cell that migrated the most during simulation time (24 h): condition $k_{ECM} = 0.0416$ N/m and $\langle\lambda\rangle^0_{FA} = 0.3$ min, setup $FA_{mat}[ON]||SF_{str}[ON]$. Link to animation:
`https://figshare.com/articles/Video_S6/12094143`

Video S7. Animation of a migrating cell displaying collective retraction (bottom view), showing cell shape and traction magnitude exerted on the substrate plane. It corresponds to cell that migrated the most during simulation time (24 h): condition

$k_{ECM} = 0.5$ N/m and $\langle\lambda\rangle^0_{FA} = 33.3$ min, setup $FA_{mat}[ON]||SF_{str}[ON]$. Link to animation: `https://figshare.com/articles/Video_S4/11604612`

Video S8. Animation of a migrating cell displaying collective retraction (bottom view), showing how front and rear lamella (Lm)(yellow) change shape within the rest of the cell surface (blue) throughout a simulation. It corresponds to cell that migrated the most during simulation time (24 h): condition $k_{ECM} = 0.5$ N/m and $\langle\lambda\rangle^0_{FA} = 33.3$ min, setup $FA_{mat}[ON]||SF_{str}[ON]$. Link to animation: `https://figshare.com/articles/Video_S5/11604621`

Video S9. Animation of a migrating cell displaying collective retraction (bottom view), showing the position presence of focal adhesions (FAs)(red) at the cell-substrate interface. It corresponds to cell that migrated the most during simulation time (24 h): condition $k_{ECM} = 0.5$ N/m and $\langle\lambda\rangle^0_{FA} = 33.3$ min, setup $FA_{mat}[ON]||SF_{str}[ON]$. Link to animation: `https://figshare.com/articles/Video_S6/11604660`

Video S10. Animation of a migrating cell displaying collective retraction (bottom view), showing the time elapsed since a stress fiber (SF) has detached from the substrate at one of its ends($\lambda_{detach,FA}$) before deletion. Collective retraction occurs when most SFs detached approximately 6 min. before. SFs are deleted approximately 15 min. after detachment. It corresponds to cell that migrated the most during simulation time (24 h): condition $k_{ECM} = 0.5$ N/m and $\langle\lambda\rangle^0_{FA} = 33.3$ min, setup $FA_{mat}[ON]||SF_{str}[ON]$. Link to animation: `https://figshare.com/articles/Video_S10/12094131`

Video S11. Animation of a migrating cell displaying collective retraction (bottom view), showing the strengthening factor ($n_{str,f}$) per stress fiber (SF) throughout a simulation. Most fibers mature fully, strengthening by a factor of 5. It corresponds to cell that migrated the most during simulation time (24 h): condition $k_{ECM} = 0.5$ N/m and $\langle\lambda\rangle^0_{FA} = 33.3$ min, setup $FA_{mat}[ON]||SF_{str}[ON]$. Link to animation: `https://figshare.com/articles/Video_S11/12095190`

Video S12. Animation of a migrating cell displaying collective retraction (bottom view), showing the time remaining for each node in the cell cortex in a refractory state (t_{ref}) before it can form a focal adhesion (FA) with the substrate below. Because rupture due to excessive force occurs for most FAs in the collective rupture event, most nodes in the rear lamellum (Lm) enter a refractory state. Those nodes in the front Lm only due so when cell is designedly detached. It corresponds to cell that migrated the most during simulation time (24 h): condition $k_{ECM} = 0.5$ N/m and $\langle\lambda\rangle^0_{FA} = 33.3$ min, setup $FA_{mat}[ON]||SF_{str}[ON]$. Link to animation: `https://figshare.com/articles/Video_S12/12095217`

Table 1: Additional parameters for model implementation

symbol	parameter	value	units	source
α	angle defining active cell areas	$\pi/4$	rad	trial runs
$k_{gen,\triangle i}$	actin generation rate	1.8e11	$1/m^2/s$	[13],[14]
k_{deg}	actin degradation rate	0.016	$1/s$	[13],[14]
D_{actin}	actin diffusion constant in cortex	8e-14	$1/s$	[13],[14]
$[G]_{Lp}$	actin threshold Lp	3.5e11	$1/m^2$	[13],[14]
$[G]_{Lm}$	actin threshold Lm	4.0e10	$1/m^2$	[13],[14]
k_{cortex}	stiffness cell cortex	2.9e-4	N/m	[15]
Λ^d	cortex damping	0.5	$N \cdot s/m$	trial runs
$k_{A,local}$	local area constraint	0.5	N/m	trial runs
$k_{A,global}$	global area constraint	1e-8	N/m	trial runs
k_{vol}	volume constraint	8.5	N/m^2	trial runs
k_{bend}	cortex bending constant	8e-16	N/m	trial runs
E_c	Young's modulus cortex	1e5	Pa	[5]
ν_c	Poisson's ratio cortex	0.4	$-$	trial runs
E_{subs}	Young's modulus substrate	1e5	Pa	trial runs
ν_{subs}	Poisson's ratio substrate	0.5	$-$	trial runs
W_{cs}	cell-substrate adhesion energy	3.334e-4	N/m	trial runs
$\lambda_{t,cs}$	cell-substrate tangential friction	10	$kPa \cdot s/m$	trial runs
$\lambda_{n,cs}$	cell-substrate normal friction	5e11	$kPa \cdot s/m$	trial runs
η_l	liquid viscocity	100	$kPa \cdot s$	trial runs

References

1. Schwarz US, Erdmann T, Bischofs IB, Focal adhesions as mechanosensors: Two-spring model, Biosystems, 83, 225-232 (2006)
2. Mitrossilis D, Fouchard J, Guiroy A, et al., Single-cell response to stiffness exhibits muscle-like behavior, PNAS, 106, 18243-8 (2009)
3. Van Gelder A, Approximate simulation of elastic membranes by triangulated spring meshes, Journal of Graphics Tools, 3, 21-41 (1998)
4. Helfrich W, Elastic properties of lipid bilayers: theory and possible experiments, Verlag der Zeitschrift für Naturforschung, 28, 693-703 (1973)
5. Odenthal T, Smeets B, Van Liedekerke P, et al., Analysis of initial cell spreading using mechanistic contact formulations for a deformable cell model, PloS Comp Biol, 9, e1003267 (2013)
6. Van Liedekerke P, Smeets B, Odenthal T, et al., Solving microscopic flow problems using stokes equations in SPH, Comp Phys Comm 184, 1686-1696 (2013)
7. Johnson KL, Greenwood JA, An adhesion map for the contact of elastic spheres. J Colloid Interface Sci, 192, 326-333 (1997)
8. Guyot Y, Smeets B, Odenthal T, et al., Immersed boundary models for quantifying flow-induced mechanical stimuli on stem cells seeded on 3D scaffolds in perfusion bioreactors, PLoS Comp Biol, 12, e1005108 (2016)
9. Guennebaud G, Benoît J, et al., Eigen v3. *http://eigen.tuxfamily.org* (2010)
10. He K, Dong S, Zhou Z, Multigrid contact detection method, Physical Review E, 75, 036710 (2007)
11. Dunavant DA, High degree efficient symmetrical Gaussian quadrature rules for the triangle, International Journal for Numerical Methods in Engineering, 21, 1129-1148 (1985)
12. Hernández-Varas P, Berge U, Lock J, et al., A plastic relationship between vinculin-mediated tension and adhesion complex area defines adhesion size and lifetime, Nature Communications, 6 (2015)
13. Delorme V, Machacek M, DerMardirossian C, et al., Cofilin activity downstream of Pak1 regulates cell protrusion efficiency by organizing lamellipodium and lamella actin networks, Dev Cell, 13, 646–662 (2007)

14. Fischer RS, Gardel M, Ma X, et al., Local cortical tension by myosin II guides 3D endothelial cell branching, Curr Biol, 19, 260-265 (2009)
15. Pontes B, Monzo P, Gauthier NC, Membrane tension: A challenging but universal physical parameter in cell biology, Semin Cell Dev Biol, 71, 30–41 (2017)
